# Cdc7-Dbf4-mediated phosphorylation of HSP90-S164 stabilizes HSP90-HCLK2-MRN complex to enhance ATR/ATM signaling that overcomes replication stress in cancer

**DOI:** 10.1038/s41598-017-17126-2

**Published:** 2017-12-05

**Authors:** An Ning Cheng, Chi-Chen Fan, Yu-Kang Lo, Cheng-Liang Kuo, Hui-Chun Wang, I.-Hsin Lien, Shu-Yu Lin, Chung-Hsing Chen, Shih Sheng Jiang, I.-Shou Chang, Hsueh-Fen Juan, Ping-Chiang Lyu, Alan Yueh-Luen Lee

**Affiliations:** 1 National Institute of Cancer Research, National Health Research Institutes, Zhunan, Miaoli, Taiwan; 2 Institute of Bioinformatics and Structural Biology, National Tsing Hua University, Hsinchu, Taiwan; 3 Superintendent Office, Mackay Memorial Hospital, Taipei, Taiwan; 4 Department of Medical Laboratory Science and Biotechnology, Yuanpei University of Medical Technology, Hsinchu, Taiwan; 5 Graduate Institute of Natural Products, College of Pharmacy, Kaohsiung Medical University, Kaohsiung, Taiwan; 6 Common Mass Spectrometry Facilities, Institute of Biological Chemistry, Academia Sinica, Taipei, Taiwan; 7 Institute of Molecular and Cellular Biology, National Taiwan University, Taipei, Taiwan; 8 Department of Life Science, National Taiwan University, Taipei, Taiwan; 9 Department of Biotechnology, Kaohsiung Medical University, Kaohsiung, Taiwan

## Abstract

Cdc7-Dbf4 kinase plays a key role in the initiation of DNA replication and contributes to the replication stress in cancer. The activity of human Cdc7-Dbf4 kinase remains active and acts as an effector of checkpoint under replication stress. However, the downstream targets of Cdc7-Dbf4 contributed to checkpoint regulation and replication stress-support function in cancer are not fully identified. In this work, we showed that aberrant Cdc7-Dbf4 induces DNA lesions that activate ATM/ATR-mediated checkpoint and homologous recombination (HR) DNA repair. Using a phosphoproteome approach, we identified HSP90-S164 as a target of Cdc7-Dbf4 *in vitro* and *in vivo*. The phosphorylation of HSP90-S164 by Cdc7-Dbf4 is required for the stability of HSP90-HCLK2-MRN complex and the function of ATM/ATR signaling cascade and HR DNA repair. In clinically, the phosphorylation of HSP90-S164 indeed is increased in oral cancer patients. Our results indicate that aberrant Cdc7-Dbf4 enhances replication stress tolerance by rewiring ATR/ATM mediated HR repair through HSP90-S164 phosphorylation and by promoting recovery from replication stress. We provide a new solution to a subtyping of cancer patients with dominant ATR/HSP90 expression by combining inhibitors of ATR-Chk1, HSP90, or Cdc7 in cancer combination therapy.

## Introduction

Cell cycle checkpoints are complex surveillance mechanisms that are used to ensure genomic stability and successful repair of DNA damage before entering next phase. ATM (ataxia telangiectasia mutated) and ATR (ataxia telangiectasia and Rad3-related protein) are two major kinases that are crucial for detecting aberrant DNA structures and activating DNA damage response (DDR)^1^. Different types of DNA lesions activate different but overlapping DDR pathways. DNA double stranded breaks (DSBs) sensed by the Mre11/Rad50/Nbs1 (MRN) complex activate the ATM-Chk2 signaling^2^; single-stranded DNAs (ssDNA) induced by replication stress activate the ATR-Chk1 signaling along with the ATRIP, HCLK2, and TopBP1^3,4^. The kinases are stimulated to transduce the signal to effector proteins that launch a cascade of events that causes cell cycle arrest, apoptosis and/or DNA repair^5^.

Replication stress is now recognized as a cancer hallmark^6,7^. Replication stress is represented by stalling of replication forks, their collapse, and DSBs formation^7^. The extent of replication stress is under the tight control of ATR-Chk1 S-phase checkpoint and DNA repair including homologous recombination (HR) repair that ensure genomic stability^4,8^. The S-phase checkpoint inhibits further firing at late origin to slow down DNA replication and prevents progression into mitosis in DDR. Unfortunately, mutations in the checkpoint or DNA repair are found in many advanced stage cancers. To cope with the increased replication stress, cancer cells bearing checkpoint- or repair-deficiency paradoxically amplify ATR-Chk1 signaling to overcome the stress for proliferation and survival^9,10^. However, how replication stress induces extensive activation of ATR-Chk1 signaling for DNA damage tolerance in cancer still is needed to explore.

Cdc7-Dbf4 kinase plays pivotal roles in the initiation of DNA replication^11,12^. Cdc7-Dbf4 also is involved in other DNA metabolism events including meiotic recombination^13^, translesion synthesis (TLS)^14–16^, and S phase checkpoint^17–19^. Initially, Cdc7-Dbf4 kinase from yeast was considered as a target of S-phase checkpoint because the activity of Cdc7-Dbf4 was inhibited upon replication stress^17,20^. However, accumulating evidences suggested that human Cdc7-Dbf4 remains active upon replication stress^18,19,21^. These conflicting findings indicate that human Cdc7-Dbf4 acts as an upstream effector to monitor S-phase checkpoint signaling. In addition, Cdc7-Dbf4 promotes checkpoint recovery, TLS, and fork protection to increase cell survival under replication stress^15,16,19,22^. Indeed, overexpression of Cdc7-Dbf4 is associated with tumor advanced clinical stage, patient survival, cell cycle deregulation, and genomic instability. We previously found that increased Cdc7 inhibits DNA damage-induced apoptosis and increases the survival of cancer cells upon DNA damage response^23^. However, the targets of human Cdc7-Dbf4 in the detailed mechanism in the coordination of checkpoint and replication stress-support function are still missing.

HSP90 is a molecular chaperone with more than 200 identified client proteins including numerous oncoproteins, such as AKT^24–26^. HSP90 plays an important role in S-phase checkpoint and DNA repair for cellular adaptation^27^. HSP90 binds the component proteins of ATR signaling, ATR^28^, MRN complex^29^ and HCLK2-TTI1/2 complex^30^. In addition, the chaperone function of HSP90 is regulated by post-translational modification^31,32^. Thus, whether the phosphorylation of HSP90 contributes to the regulation of S-phase checkpoint and DNA repair is still unknown. In this paper, we unveiled a novel role of Cdc7-Dbf4 in the regulation of ATR/ATM checkpoint signaling and HR DNA repair in cancer by phosphorylating HSP90 at S164.

## Results

### Overexpression of Cdc7 induces replication that causes DNA DSB and activates ATM/ATR-mediated checkpoint

**Figure 1:**
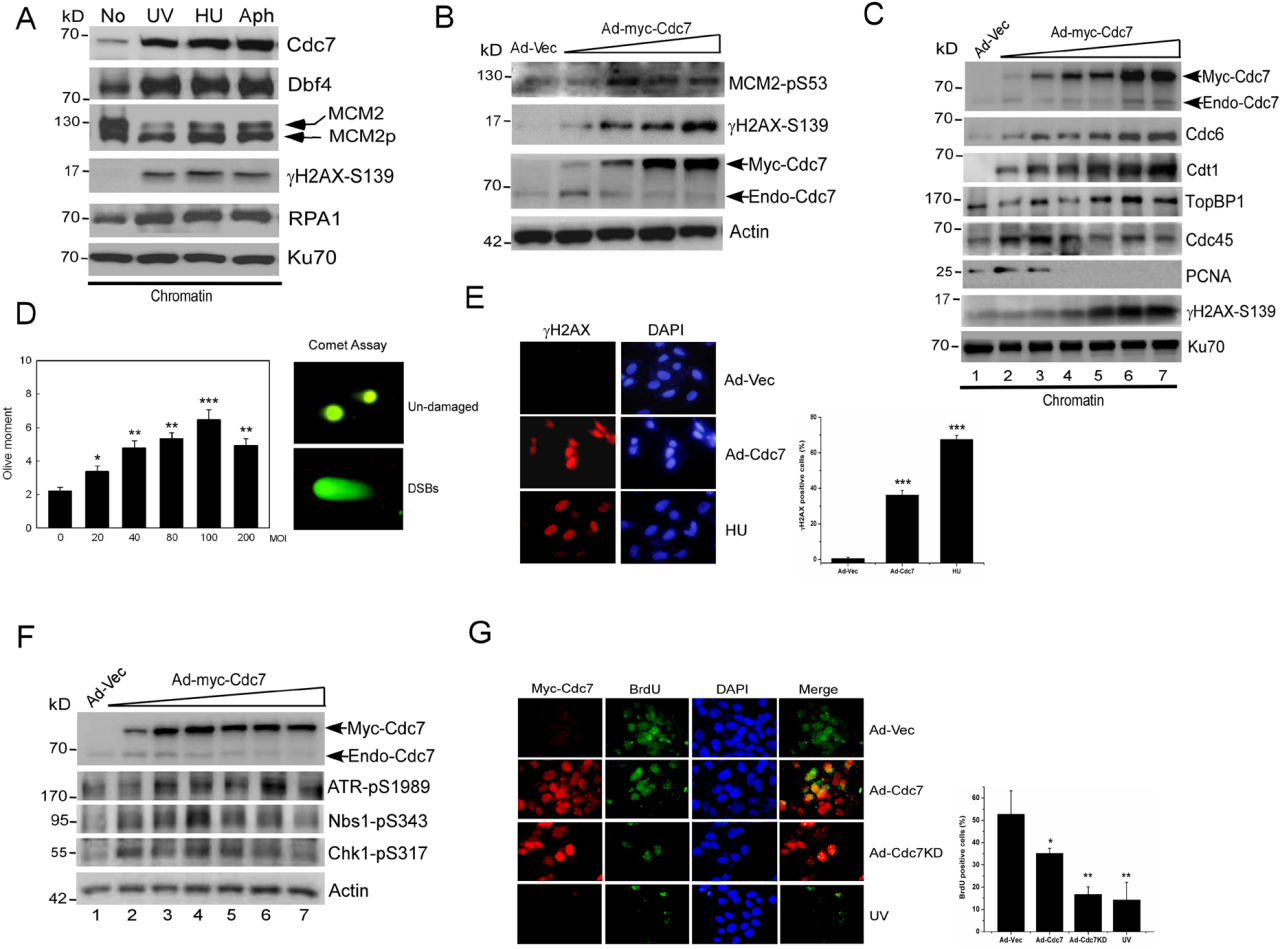
Cdc7 overexpression causes DNA lesions and activates ATM/ATR-mediated checkpoint. (**A**) Cdc7 and Dbf4 accumulate on stalled fork to activate more MCM2 phosphorylation and cause the formation of DNA lesions upon replication stress. Chromatin fractions were isolated from U2OS cells after mock treatment (No), or UV (50 J/m^2^), HU (1 mM, 24 h after), or Aphidicolin (Aph, 1 μg/ml, 24 h after) and were applied to immunoblotting. Equal amounts of input protein were subjected to immunoblotting using anti-Cdc7, anti-Dbf4, anti-MCM2, anti-γ-H2AX, and anti-RPA1 antibodies. Ku70 was served as a loading control. (**B**) Cdc7 overexpression induces DSB formation. U2OS was infected with Ad-Cdc7 (0.6, 1.2, 2.4, or 3.6 × 10^8^ pfu/ml) or Ad-Vec (3.6 × 10^8^ pfu/ml) for 48 h. The lysates were analyzed by Western blotting using the indicated antibodies. The expression of endogenous Cdc7 (Endo-Cdc7) and adenovirally introduced Myc tagged Cdc7 (Myc-Cdc7) is shown as indicated. (**C**) Cdc7 overexpression triggers the initiation of DNA replication and causes DSB formation that activates S-checkpoint. U2OS was infected with Ad-Cdc7 (0.3, 0.6, 1.2, 2.4, 3.6, or 7.2 × 10^8^ pfu/ml) or Ad-Vec (3.6 × 10^8^ pfu/ml) for 48 h. Chromatin fractions were isolated and analyzed by immunoblotting. Equal amounts of input protein were subjected to immunoblotting using anti-Cdc7, anti-Cdc6, anti-Cdt1, anti-TopBP1, anti-Cdc45, anti-PCNA, and anti-γ-H2AX antibodies. Ku70 was served as a loading control. The expression of endogenous Cdc7 (Endo-Cdc7) and adenovirally introduced Myc tagged Cdc7 (Myc-Cdc7) is shown. (**D**) Comet assays indicate DSB formation when Cdc7 is overexpressed. U2OS was infected with Ad-Cdc7 using different viral titers (0.6 × 10^8^ pfu/ml, MOI = 20; 1.2 × 10^8^ pfu/ml, MOI = 40; 2.4 × 10^8^ pfu/ml, MOI = 80; 3.6 × 10^8^ pfu/ml, MOI = 100; 7.2 × 10^8^ pfu/ml, MOI = 200) or Ad-GFP control. Olive moment for each condition was measured using the Comet Score software (TriTek Corporation, Sumerduck, VA, USA). Fifty cells were counted for each experiment. Two typical cells with or without comet tails are shown on the right. Results are presented as range of three independent experiments. *P < 0.05, **P < 0.01, ***P < 0.001. (**E**) Cdc7 overexpression triggers γH2AX formation by immunofluorescence analysis. U2OS cells were infected with Ad-Cdc7 (2.4 × 10^8^ pfu/ml) or Ad-Vec for 48 h and immunostaining was performed by using antibodies toward γH2AX. U2OS cells treated with hydroxyurea (HU, 1 mM) for 16 h only were used as a positive control. DAPI was used for nuclear staining. The graph with error bars is shown in the right panel. Results are presented as range of three independent experiments. *P < 0.05, **P < 0.01, ***P < 0.001. (**F**) Cdc7 overexpression activates ATR-mediated checkpoint. U2OS was infected with Ad-Cdc7 (0.3, 0.6, 1.2, 2.4, 3.6, or 7.2 × 10^8^ pfu/ml) or Ad-Vec (3.6 × 10^8^ pfu/ml) for 48 h. The lysates were analyzed by immunoblotting using anti-Cdc7, anti-phospho-ATR (S1989), anti-phospho-Nbs1 (S343), and anti-phospho-Chk1 (S317) antibodies. β-actin was served as a loading control. The expression of endogenous Cdc7 (Endo-Cdc7) and adenovirally introduced Myc tagged Cdc7 (Myc-Cdc7) is shown. (G) Cdc7 overexpression reduces BrdU incorporation. U2OS cells were infected with Ad-Cdc7 (2.4 × 10^8^ pfu/ml), Ad-Cdc7KD (2.4 × 10^8^ pfu/ml), or Ad-Vec (2.4 × 10^8^ pfu/ml) for 48 h, and immunostaining was performed by using antibodies toward BrdU and Myc. U2OS cells treated with UV irradiation (50 J/m^2^) only were used as a positive control. DAPI was used for nuclear staining. The graph with error bars indicating the standard error from the average is shown in the right panel. Results are presented as range of three independent experiments. *P < 0.05, **P < 0.01, ***P < 0.001.

Cdc7 and Dbf4 are overexpressed in many types of cancer including oral cancer^23,33^. To explore how increased Cdc7-Dbf4 contributes to DNA damage tolerance that contributes to tumorigenesis, we tried to solve the puzzle by triggering Cdc7 overexpression in cells. First, Cdc7 and Dbf4 accumulated on stalled fork and caused more MCM2 phosphorylation when cells were treated with replication stress agents, UV, HU or aphidicolin (Fig. 1A). RPA1, a single-stranded DNA (ssDNA)-binding protein, and γH2AX, a marker of double-stranded breaks (DSBs), were also accumulated on the damaged DNA (Fig. 1A). This result suggests that Cdc7-Dbf4 is activated and correlated with ssDNA/DSB formation under replication stress. We used the adenovirus expressing Cdc7 (Ad-myc-Cdc7) to increase Cdc7 activity and Cdc7 kinase dead mutant (Ad-myc-Cdc7KD) to inhibit Cdc7 activity in cells, which was confirmed by the status of phosphorylation of MCM2 at S53 or S40/S41 (Fig. 1B and Supplementary Figure 1A) and the Cdc7-dependent chromatin loading of initiation factor Cdc45 (Supplementary Figure 1B). As expected, the cells expressing excess Cdc7 showed a strong positive γH2AX signal in a dose-dependent manner (Fig. 1B,C), which was confirmed by the comet assay (Fig. 1D) and immunofluorescence staining (Fig. 1E and Supplementary Figure 2A). Furthermore, increased Cdc7 activity promoted the accumulation of Cdc6, Cdt1, and TopBP1 on chromatin in a dose-dependent manner but inhibited the chromatin binding of Cdc45 and PCNA when Cdc7 was highly expressed (Fig. 1C). This result suggests that overexpressed Cdc7-Dbf4 always augments pre-replicative complex (pre-RC) assembly of replication but represses origin firing of replication because Cdc7 overexpression activates checkpoint. These results indicate that increased Cdc7 activity leads to the formation of DSBs that activates S-phase checkpoint. Indeed, increased Cdc7 promotes the activation of ATR-mediated S-phase checkpoint (Fig. 1F). We confirmed the S-checkpoint activation by the extent of BrdU incorporation when Cdc7 was overexpressed. The BrdU positive cells were reduced when Cdc7 was overexpressed and were reduced more by Ad-Cdc7KD, and UV treatment was a positive control (Fig. 1G). Consistently, FACS analysis also showed that overexpressed Cdc7 inhibits the signal of BrdU and increases γH2AX-positive cells after double staining with BrdU and γH2AX in the cells overexpressing Cdc7 (Supplementary Figure 2B). These data showed that increased Cdc7 upregulates the initiation of DNA replication, but excess Cdc7 causes DSB formation that activates S-phase checkpoint signaling.

### Overexpression of Cdc7 initially activates S-phase checkpoint and delays cell cycle for DNA repair and then promotes recovery from replication stress

**Figure 2:**
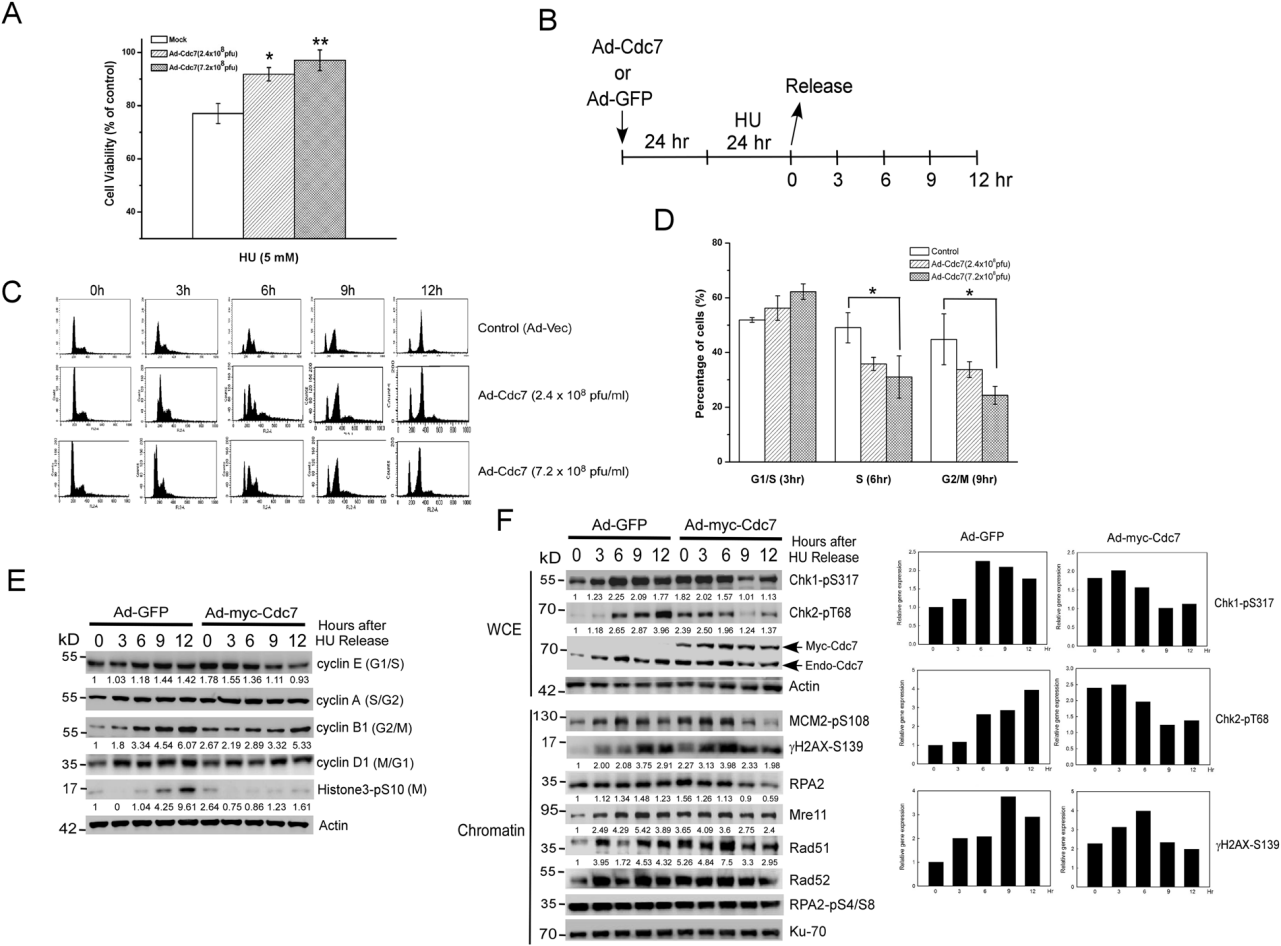
Aberrant Cdc7 activates S-phase checkpoint and homologous recombination (HR) DNA repair during recovery from replication stress. (**A**) Cdc7 overexpression reinforces cancer cells survival in response to HU treatment. U2OS cells infected with adenovirus encoding GFP (Mock, Ad-Vec, 7.2 × 10^8^ pfu/ml) or Myc-Cdc7 (Ad-Cdc7, 2.4 or 7.2 × 10^8^ pfu/ml) were incubated for 24 hours and then the cells were treated by hydroxyurea (HU, 5 mM). After 24 hour treatment, cell viability was determined. (**B**) Treatment protocol for HU release experiment. U2OS cells infected with Ad-Cdc7 (2.4 or 7.2 × 10^8^ pfu/ml) or Ad-GFP (7.2 × 10^8^ pfu/ml) for 24 hours were treated with 2 mM HU for 24 hours, and the treated cells were released into normal medium for the time indicated. C and D. Cdc7 overexpression delays cell cycle progression after HU treatment. Infected U2OS cells were treated with 2 mM HU for another 24 hours, released from the treatment, and collected at indicated time. FACS analysis was performed to demonstrate cell cycle distribution (**C**) and the percentages of cells in G1/S (3 hr), S (6 hr) and G2/M (9 hr) phase are shown in (**D**). E and F. Cdc7 overexpression activates S-phase checkpoint, delays cell cycle progression, and promotes HR repair after HU treatment. U2OS cells infected with Ad-GFP or Ad-myc-Cdc7 (7.2 × 10^8^ pfu/ml) were released from HU treatment (2 mM for 24 hrs). The cell lysates from different time points after the treatment were collected as whole cell extract (WCE) and were also used to separate the Triton-insoluble chromatin-containing fraction (chromatin). The samples were analyzed by immunoblotting. Equal amounts of input protein were subjected to immunoblotting using anti-cyclin E, anti-cyclin A, anti-cyclin B1, anti-cyclin D1, anti-Histon3-pS10, anti-Chk1-pS317, anti-Chk2-pT68, anti-MCM2-pS108, anti-γH2AX-pS139, anti-RPA2, anti-Mre11, anti-Rad51, anti-Rad52, and anti-RPA2-pS4/8 antibodies. β-actin and Ku70 were served as a loading control. The number represents the band intensity normalized against β-actin or Ku70. Histograms of anti-Chk1-pS317, anti-Chk2-pT68, and anti-γH2AX-pS139 level are shown in the right panel.

S-phase checkpoint is considered as a barrier to tumorigenesis because it prevents DNA damage from transmitting to daughter cells. To study the conflicting observation on tumorigenesis that overexpressed Cdc7 activates S-phase checkpoint and DNA damage tolerance, U2OS overexpressing Cdc7 cells were treated with HU and observed the effect on cell proliferation and cell cycle. The cell viability of U2OS cells overexpressing Cdc7 was significantly higher than control cells treated with HU (Fig. 2A), which is consistent with previous finding that upregulated Cdc7 protects cells from DNA damage agents^23^. To understand the effect of Cdc7 overexpression on checkpoint regulation, the HU-treated cells were released from the replication stress under Cdc7 overexpression or not (Fig. 2B). The results showed that Cdc7-overexpressed cells were arrested in G1/S phase (3 hr) and delayed to enter S (6 hr) and M phase (9 hr) in a dose-dependent manner (Fig. 2C–E, and Supplementary Figure 3). In addition, when Cdc7 was overexpressed, ssDNA (RPA2) accumulation on chromatin and the phosphorylation of Chk1, Chk2, MCM2-S108, and γH2AX were increased during the first 6 hr after HU release (Fig. 2F, Ad-myc-Cdc7 set, 0 to 6 hr). These data confirmed that overexpression of Cdc7 causes DNA lesions (ssDNA and DSB) and activates S-phase checkpoint. Interestingly, we also found that HR DNA repair proteins, RPA2, Mre11, Rad51, and Rad52, were increased on damaged DNA sites (Fig. 2F, 0 to 6 hr). However, non-homologous end-joining (NHEJ) pathway, RPA2 phosphorylation at S4/S8^34^, is not involved in Cdc7-induced DNA repair (Fig. 2F). After 6 hr from release, Cdc7 overexpression reduced the phosphorylation of H2AX, checkpoint proteins (e.g. Chk1 and Chk2), and the HR proteins (e.g. RPA2 and Rad51) on damaged DNA (Fig. 2F, Ad-myc-Cdc7 set, 9 to 12 hr). These results suggest that overexpression of Cdc7 initially activates checkpoint and delays cell cycle progression, and it then induces HR repair and recovery from replication stress.

**Figure 3:**
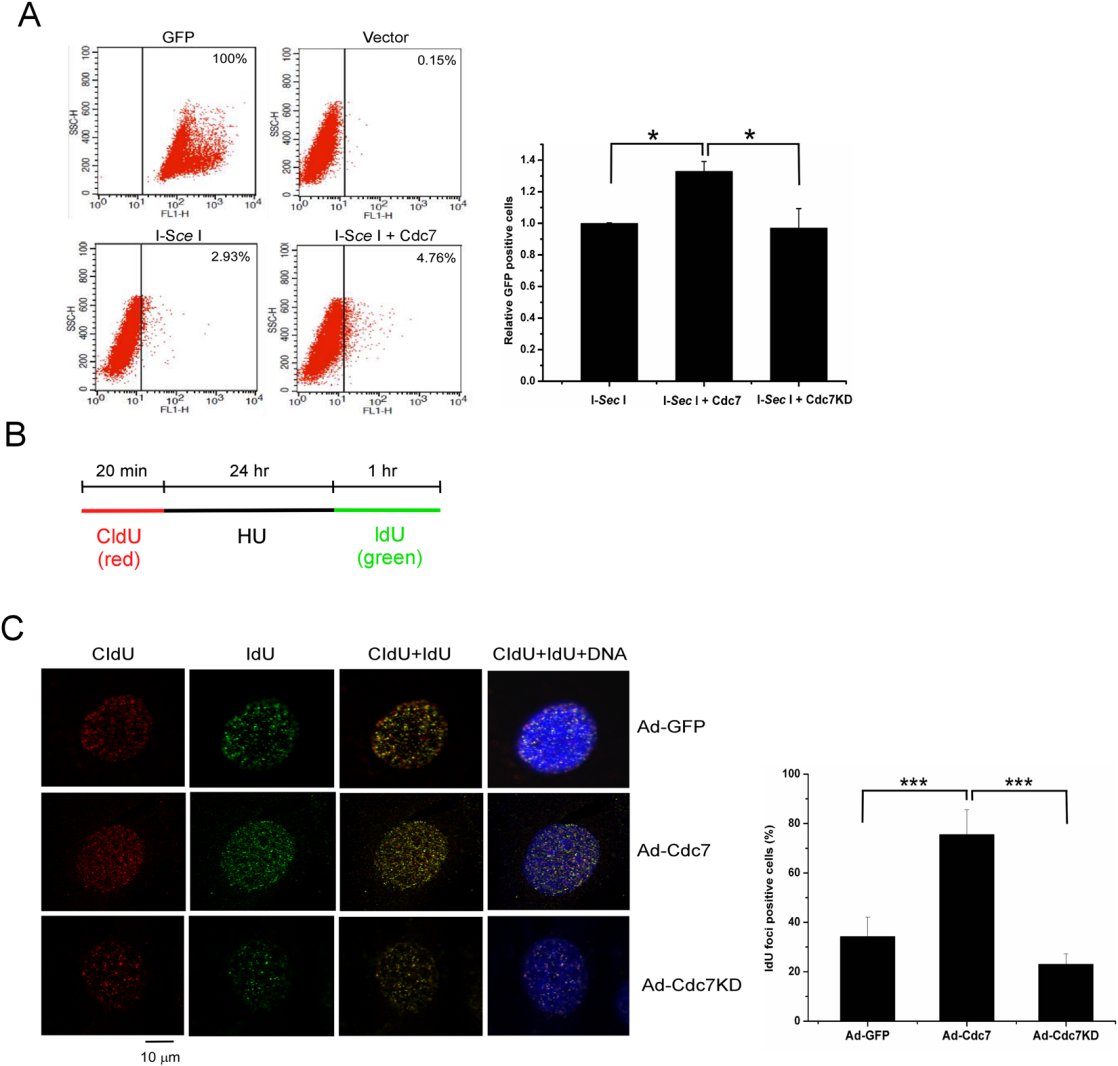
Cdc7 overexpression promotes homologous recombination (HR) DNA repair and replication restart under replication stress. (**A**) Upregulated Cdc7 facilitates HR-mediated DSB DNA repair. U2OS (DR-GFP) cells were transfected with vector only, I-Sce1, or co-transfected with myc-Cdc7 or myc-Cdc7KD. After transfecting I-Sce1 endonuclease, iGFP was used as a template for HR DSB repair. The efficiency of DSB repair was determined by the cells carrying an intact DR-GFP. The percentage of GFP-positive cells was determined by flow cytometry and shown in the left panel. The relative amount of GFP-positive cells was determined by the ratio of the experimental value and the I-Sce1 only and shown in the right panel. The error bars represent the standard deviation from three different samples. U2OS cells were transfected with GFP as a positive control, while U2OS (DR-GFP) cells with vector only were as a negative control. (**B**) Labeling protocol for DNA replication foci analysis. U2OS cells infected with Ad-Cdc7 (7.2 × 10^8^ pfu/ml) or Ad-GFP for 24 hours were pulse-labeled with CldU, treated with 2 mM HU, and released into IdU for the indicated time. CldU and IdU were detected using specific antibodies in red and green, respectively. C. Cdc7 overexpression promotes global replication restart after release from HU treatment. Quantification of cells newly entering S phase after release from 24 hr HU treatment. U2OS cells were infected, pulse-labeled, and prepared as in (**B**), then fixed and immunostained by CldU (red) and IdU (green). DNA was counterstained with DAPI (blue). The infected cells labeled with only IdU are shown as percentages of total cells. The means and standard deviation (SD) (bars) of at least three independent experiments are shown. Values marked with asterisks are significantly different (Student’s t test, ***p < 0.001).

### Overexpression of Cdc7 promotes HR DNA repair and replication restart under replication stress

We next proved whether upregulated Cdc7 promotes recovery and HR DNA repair after fork stalling. We first performed the HR DSB repair assay using I-SceI endonuclease to introduce genomic DSBs in U2OS cells that are transfected by DR-GFP reporter containing two mutated GFP cassettes, SceGFP and iGFP^35^. The efficiency of HR repair was measured 48 hours later by scoring the percentage of GFP-positive cells in the I-SceI-transfected cells. The percentage of GFP-positive cells was significantly higher in Cdc7-overexpressing cells compared with the cells without Cdc7 overexpression, and this increase was inhibited by overexpression of Cdc7KD mutant (Fig. 3A). This data indicates that Cdc7 overexpression stimulates HR DNA repair. Since Cdc7 kinase is crucial to the initiation of DNA replication, we analyzed whether Cdc7 overexpression promotes replication restart after HU treatment by using replication foci analysis^36^. U2OS cells were pulse-labeled with 5-chlorodeoxyuridine (CldU), treated with HU for 24 hr, and pulse-labeled with 5-iododeoxyuridine (IdU) as indicated (Fig. 3B). Overexpression of Cdc7 but not Cdc7KD significantly increased cell numbers of only labeled with IdU, which presents the newly-entered S phase cells (Fig. 3C). The results indicate that overexpression of Cdc7 promotes HR DNA repair, replication restart, and new origin firing during recovery from replication stress.

**Figure 4:**
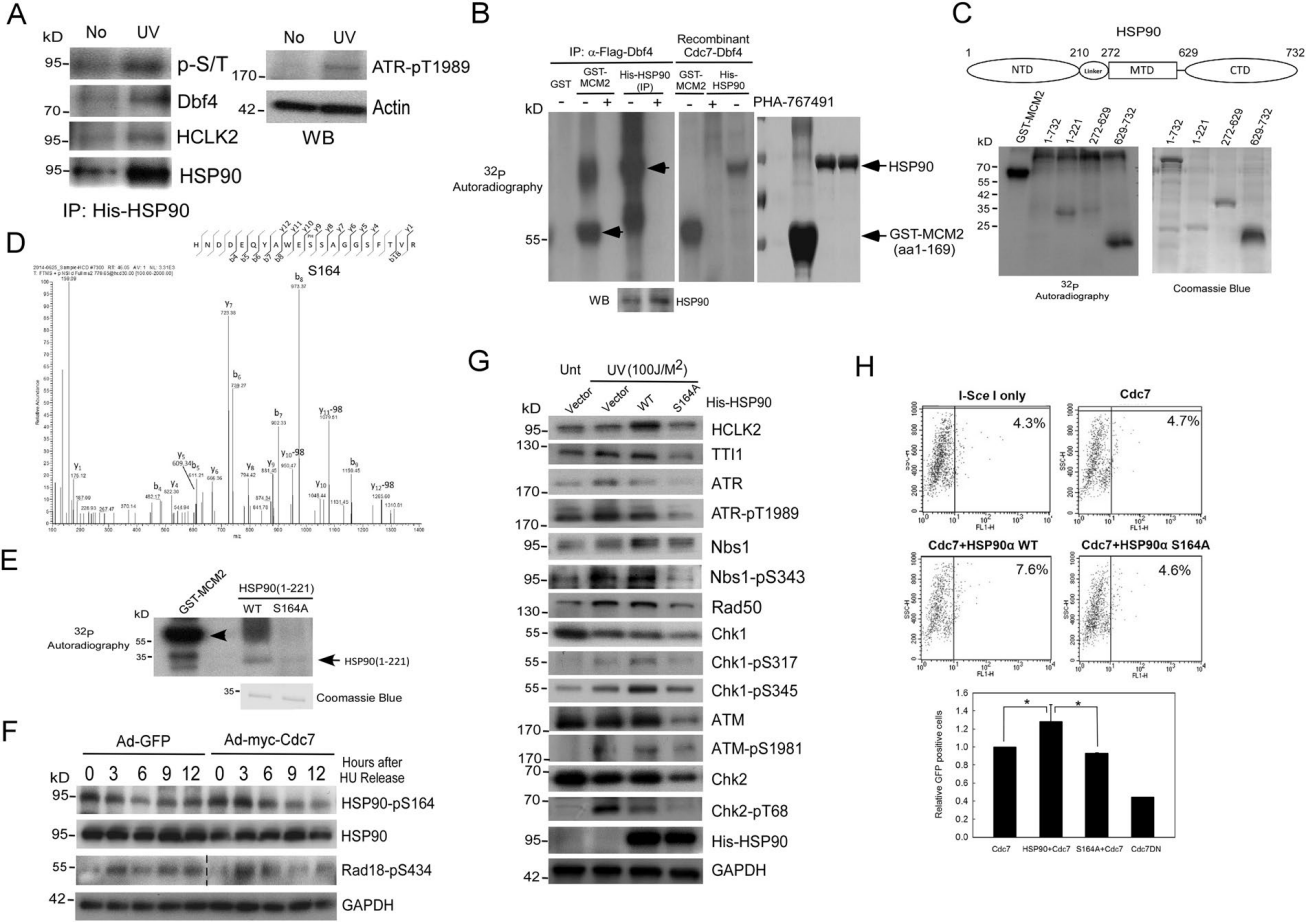
Cdc7-Dbf4 phosphorylates HSP90α at Ser-164 and the phosphorylation is required for the activation of ATM/ATR-mediated checkpoint and HR repair. (**A**) HSP90α is a phosphorylated protein upon UV treatment. The plasmid encoding His-HSP90 was transfected into 293T cells followed by treatment with UV (50 J/m^2^). The cell lysates were collected and analyzed by immunoprecipitation and immunoblotting. Equal amounts of input protein were subjected to immunoblotting using anti-phospho-Serine/anti-phospho-Threonine (p-S/T), anti-Dbf4, anti-HCLK2, anti-HSP90, and anti-ATR-pT1989 antibodies. β-actin was served as a loading control. The phosphorylation of ATR-T1989 was a positive control to UV treatment (right). (**B**) Cdc7-Dbf4 phosphorylates HSP90α *in vitro*. *In vitro* kinase assay was performed using 293T cells that is transiently transfected with FLAG-tagged Dbf4 and Cdc7 plasmid. Anti-FLAG immunoprecipitates were incubated with the pulled-down His-HSP90α or GST-MCM2 (aa1-169) as a positive control in the presence of [γ-^32^P]ATP at 30 °C for 40 mins. Before incubation, each set of immunoprecipitates was treated with or without PHA-767491. Phosphorylation of HSP90α and the input of immunoprecipitated HSP90α were shown by autoradiogram (*left*) and Western blotting (bottom), respectively. Recombinant Cdc7-Dbf4 was also used to incubate with recombinant His-HSP90α or GST-MCM2 (aa1-169) in the presence of [γ-^32^P]ATP. Phosphorylation of HSP90α and the input of GST-MCM2 and recombinant HSP90α were shown by autoradiogram (*middle*) and Coomassie Blue-stained gel (*right*), respectively. (**C**) Cdc7-Dbf4 phosphorylates N- and C-terminal domain in HSP90α *in vitro*. *In vitro* kinase assay was performed using 293T cells that is transiently transfected with FLAG-tagged Dbf4 and Cdc7 plasmid as described in (B). Cdc7-mediated phosphorylation of HSP90α fragments was revealed by an ^32^P autoradiogram (*left*), and relative input for the His-fused HSP90α fragments is shown by a Coomassie Blue-stained gel (*right*). D and E. Cdc7-Dbf4 phosphorylates HSP90α at Ser 164. (**D**) Purified Cdc7-Dbf4 and HSP90α were incubated at 30 °C for 40 mins, then the mixture was collected after in-gel digestion and used in MS/MS spectrum analysis. NanoLC−nanoESI-MS/MS analysis was performed on a nanoAcquity system. MS/MS spectrum shows [M + 2 H]^2+^ (m/z 779.65) ion of the peptide HNDDEQYAWE[pS]SAGGSFTVR from the HSP90α protein, which is a phosphorylated peptide. (**E**) *In vitro* kinase assay was performed. Anti-FLAG immunoprecipitates were incubated with the purified His-HSP90α fragment (1-221) WT or S164A mutant in the presence of [γ-^32^P]ATP at 30 °C for 40 mins. The phosphorylation of HSP90α fragments was revealed by an ^32^P autoradiogram (*top*), and relative input for the His-HSP90α fragments is shown by a Coomassie Blue-stained gel (*bottom*). (**F)** Cdc7 overexpression promotes the recovery from HU treatment by phosphorylating HSP90α-S164 and Rad18-S434 *in vivo*. U2OS cells infected with Ad-GFP or Ad-myc-Cdc7 (7.2 × 10^8^ pfu/ml) were released from HU treatment (2 mM for 24 hrs). The cell lysates from different time points after the treatment were collected as whole cell extract. The samples were analyzed by Western blotting using the indicated antibodies. (**G**) HSP90α-S164 phosphorylation is required for the stability and activation of the ATM/ATR signaling complex upon UV exposure. U2OS cells transfected with the plasmid encoding HSP90α-WT and S164A mutant were exposed to UV (100 J/m^2^) and incubated for 3 hours. Cell lysates were prepared and immunoblotting was performed using the indicated antibodies. (**H**) HSP90α-S164 phosphorylation is required for the activation of HR DNA repair. I-Sce1-transfected U2OS cells were co-transfected with the plasmids encoding Cdc7 only or Cdc7 plus HSP90α-WT or S164A mutant. The efficiency of HR repair was measured 48 hours later by scoring the percentage of GFP-positive cells in the I-Sce1-transfected cells. The relative amount of GFP-positive cells was determined by the ratio of the experimental value and Cdc7 only and shown in the bottom panel. The error bars represent the standard deviation from three different samples.

### The phosphorylation of HSP90-Ser164 by Cdc7-Dbf4 is required for the maintenance of ATM/ATR-mediated checkpoint activation and HR DNA repair under replication stress

Since Cdc7-Dbf4 is active under replication stress and plays a role in ATM/ATR checkpoint and HR DNA repair, we attempted to identify the targets of human Cdc7-Dbf4 under replication stress by using a phosphoproteomic experiment. By phosphoproteome analysis, we acquired 831 unique phosphopeptides corresponding to 1209 quantified phosphosites on 462 phosphoproteins including TOP2A^37^, MCM2^38^, Cdt1^39^, HSP90 (Table S1-S3). We indeed found that the HSP90-HCLK2 complex was phosphorylated after UV treatment (Fig. 4A). To validate HSP90 is a direct substrate of Cdc7-Dbf4 kinase, we performed *in vitro* kinase assay using different approaches. *In vitro* kinase assays showed that HSP90 is phosphorylated (about 95 kDa) when His-HSP90 was immunoprecipitated from 293T cells as an *in vitro* substrate (Fig. 4B, left) and GST-MCM2 (1-169 aa) as a control substrate^19^. Consistently, HSP90 phosphorylation was dramatically inhibited when Cdc7 inhibitor (PHA767491) was used (Fig. 4B, left). We also confirmed that Cdc7-Dbf4 directly phosphorylates HSP90 using recombinant Cdc7-Dbf4 as a kinase and recombinant His-HSP90 as a substrate (Fig. 4B, right). *In vitro* kinase assay showed that the N-domain (1-221 residues) and C-domain (629-732 residues) of HSP90α were phosphorylated by Cdc7-Dbf4 (Fig. 4C). Furthermore, we identified the Cdc7-Dbf4 phosphorylation sites of HSP90α at S164 (Fig. 4D) and S263 (Supplementary Figure 4) by mass spectrometry. The phosphorylation site of HSP90α at Ser164 was confirmed by the introduction of HSP90α-S164A mutation (Fig. 4E). To prove HSP90 phosphorylation at Ser164 *in vivo*, we generated a phospho-specific antibody for S164 phosphorylation of HSP90α, and the specificity of affinity-purified antibody was confirmed (Supplementary Figure 5). To verify the function of HSP90α phosphorylation by Cdc7-Dbf4 in checkpoint regulation *in vivo*, we repeated the HU release experiment shown in Fig. 2B. We found that the S164 phosphorylation of HSP90α was increased during 0 to 3 hr and decreased after 6 hr (Fig. 4F). Taken together, these results confirmed that Cdc7-Dbf4 phosphorylates HSP90 at Ser164 *in vitro* and *in vivo*. Intriguingly, we also observed that the trend in Rad18 phosphorylation at S434 is similar to the one in HSP90α S164 phosphorylation (Fig. 4F). Cdc7-Dbf4 phosphorylates Rad18 to guide DNA polymerase η to sites of stalling fork and to activate Rad18 to mono-ubiquitinate PCNA, which promotes TLS^15^. This result indicates that increased Cdc7-Dbf4 also promotes DNA damage tolerance under replication stress through activating TLS.

**Figure 5:**
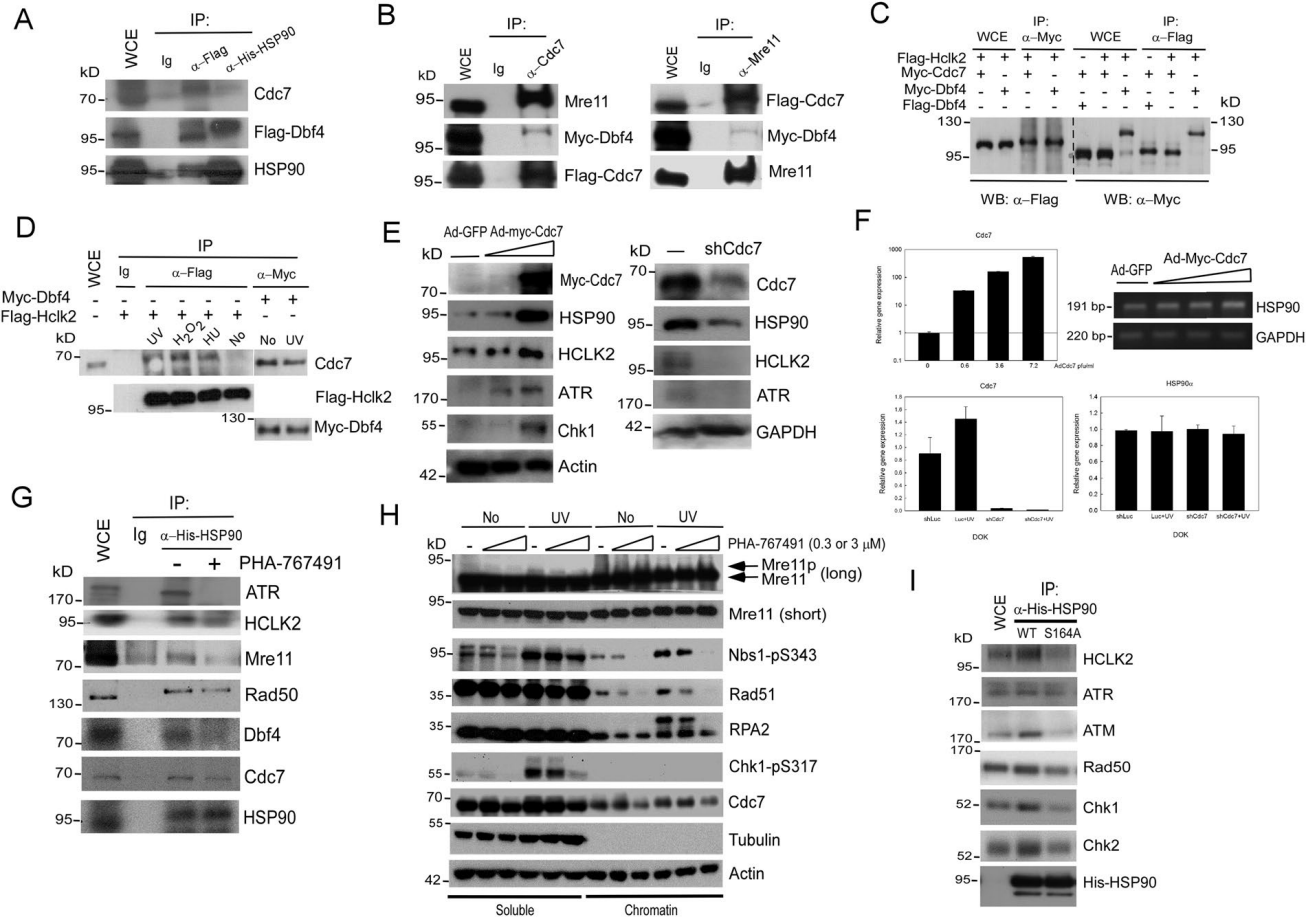
The phosphorylation of HSP90-Ser164 by Cdc7-Dbf4 is required for the stability of ATR-HSP90-HCLK2-Mre11 complex that supports HR DNA repair and recovery under replication stress. (**A**) Cdc7-Dbf4 interacts with HSP90α.The plasmids encoding Flag-tagged Dbf4 was cotransfected with plasmid encoding His-HSP90α into 293T cells. Immunoprecipitation and immunoblotting were performed as indicated antibodies. (**B**) Cdc7-Dbf4 interacts with Mre11. 293T cells were co-transfected with plasmids encoding Flag-tagged Cdc7, Myc-tagged Dbf4, and Mre11 followed by co-immunoprecipitation with anti-Cdc7 and anti-Mre11 antibody, respectively. Immunoprecipitation and immunoblotting were performed as indicated. (**C**) Cdc7-Dbf4 interacts with HCLK2. The plasmids encoding Myc-Cdc7, Flag-tagged Dbf4, or Myc-tagged Dbf4 were cotransfected with plasmid encoding Flag-HCLK2 into 293T cells. Immunoprecipitation and immunoblotting were performed as indicated. (**D**) The association between Cdc7-Dbf4 and HCLK2 increases in response to DNA damage stress. The plasmids encoding Myc-tagged Dbf4 were cotransfected with plasmid encoding Flag-HCLK2. The transfected cells were treated with or without UV radiation (100 J/m^2^), hydrogen peroxide (200 μM), or HU (2 mM). Immunoprecipitation was performed as indicated. (**E**) Cdc7 is important for the stability of HSP90, HCLK2, and ATR proteins. For overexpression experiment, U2OS cells was infected with Ad-Cdc7 using different viral titers (0.6 × 10^8^ pfu/ml or 3.6 × 10^8^ pfu/ml) or Ad-GFP control (3.6 × 10^8^ pfu/ml). For knocking-down experiment, Cdc7 expression was inhibited by Cdc7-shRNA transfection. Immunoblotting were performed using the indicated antibodies. (**F**) The level of Cdc7 does not affect the gene expression of HSP90 and HCLK2 in RNA level. Cells were infected with Ad-Cdc7 using different viral titers (0.6, 3.6, or 7.2 × 10^8^ pfu/ml) for overexpression or transfected with Cdc7-shRNA for down-regulation. Afterwards, cells were collected and extracted to obtain RNA that was used to reverse-transcribe to cDNA. Quantitative real-time PCR analysis or DNA gel electrophoresis showed the RNA level of HSP90. The error bars shown in the panel represent the standard deviation from three different experiments. (**G**) The activity of Cdc7-Dbf4 is required for the binding between HSP90 and checkpoint proteins including ATR, HCLK2, MRN, and Cdc7-Dbf4. 293T cells were transfected with plasmid encoding His-HSP90α and treated with or without PHA-767491 (5 μM) for 3 h. Immunoprecipitation and immunoblotting were performed using the indicated antibodies. (**H**) The activity of Cdc7-Dbf4 is required for chromatin-unbound Mre11 phosphorylation and the recruitment of HR repair proteins from upon UV stress. U2OS cells were treated with PHA-767491 (0.3 or 3 µM) for 6 h and treated with or without UV radiation (100 J/m^2^) followed by recovery for 2 h. The soluble and chromatin fraction were analyzed by immunoblot using the indicated antibodies. (**I**) HSP90-S164 phosphorylation is required for the interaction of HSP90 and checkpoint proteins including ATR, ATM, HCLK2, Rad50, Chk1 and Chk2. 293T cells were transfected with plasmids encoding His-tagged HSP90 WT or S164A. Immunoprecipitation and immunoblotting were performed using the indicated antibodies.

Since HSP90-HCLK2 complex is an important activator to ATR signaling, we examined whether the S164 phosphorylation of HSP90 regulates the ATM/ATR activation. We found that HSP90α-S164A mutant decreased the stability of the ATM/ATR signaling complex including ATM, ATR, HCLK2, TTI1, and MRN complex and attenuated the ATM/ATR signaling upon UV exposure (Fig. 4G). Consistently, overexpression of HSP90 increased the HR DNA repair caused by Cdc7, but the S164A mutant inhibited the HR DNA repair (Fig. 4H). These results indicate that the phosphorylation of HSP90-Ser164 by Cdc7-Dbf4 is required for the maintenance of ATM/ATR-mediated S-phase checkpoint activation, HR DNA repair, and the stability of ATM/ATR signaling component proteins under replication stress.

### The phosphorylation of HSP90-Ser164 by Cdc7-Dbf4 is required for the stability of ATR-HSP90-HCLK2-Mre11 complex that supports HR DNA repair and recovery under replication stress

We further confirmed whether Cdc7-Dbf4 regulates ATR/ATM signaling via direct stabilizing ATR/ATM activating auxiliary proteins, HCLK2, HSP90, and Mre11. We first confirmed that HCLK2 is required for ATR signaling activation, including the phosphorylation of Chk1 at S317 and Dbf4 at S539 (Supplementary Figure 6). Consistently, Cdc7-Dbf4 indeed interacts with HSP90 (Fig. 5A) and Mre11 (Fig. 5B), and HCLK2 (Fig. 5C) shown by reciprocal co-immunoprecipitation. The association with HCLK2 is enhanced by UV, H_2_O_2_, or HU treatment (Fig. 5D). Cdc7-Dbf4 and HSP90 interact with the N- and C-terminal domain of HCLK2 (Supplementary Figure 7). We found that the protein level of HSP90, HCLK2, and ATR was increased in Cdc7-overexpressing cells and decreased when Cdc7 was knocked-down (Fig. 5E), suggesting that Cdc7-Dbf4 contributes to stabilize HSP90-ATR-HCLK2 complex. However, the RNA level of HCLK2 and HSP90 has no significant difference in spite of Cdc7 level (Fig. 5F). The inhibition of Cdc7-Dbf4 activity by PHA-767491 affects the interaction between HSP90 and ATR, HCLK2, MRN complex, as well as HSP90 and Cdc7-Dbf4 (Fig. 5G). In addition, inhibition of Cdc7 activity reduced ATM/ATR-dependent chromatin-unbound Mre11 phosphorylation and the recruitment of HR repair proteins on chromatin (Fig. 5H), suggesting that the activity of Cdc7-Dbf4 is important for ATR-HSP90-mediated DNA repair and subsequent recovery following replication stress^40^. Importantly, the HSP90-S164A mutant decreased the interaction between HSP90 and ATM/ATR as well HCLK2 (Fig. 5I). These results show that the activity of Cdc7-Dbf4 is required for DNA repair and subsequent recovery under replication stress, which is accomplished by binding/stabilizing ATR-HSP90-HCLK2-Mre11 complex through HSP90-S164 phosphorylation.

**Figure 6:**
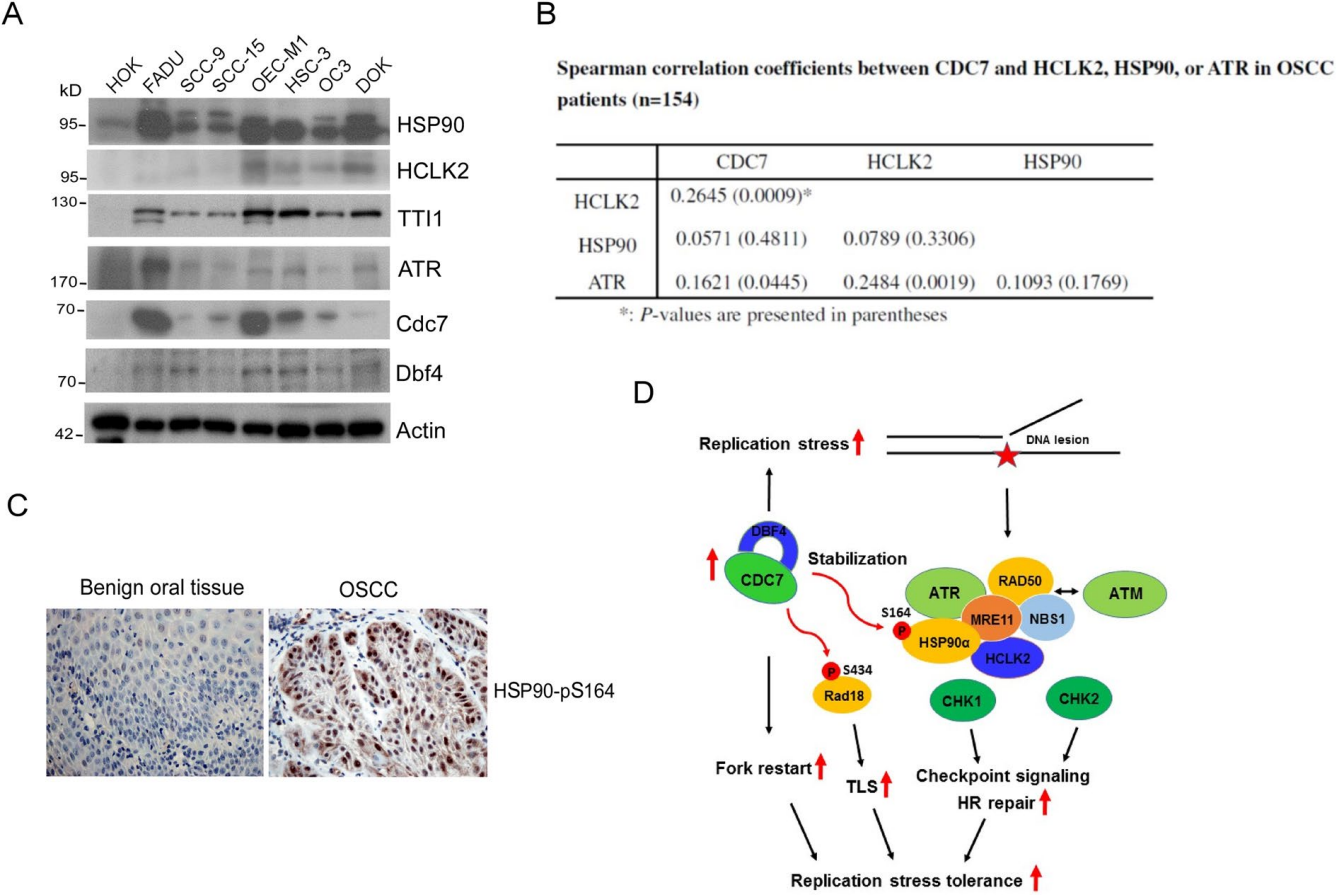
Clinical significance of Cdc7-ATR-HCLK2 complex and the phosphorylation of HSP90α at Ser-164 in oral squamous cell carcinoma (OSCC). (**A**) Aberrant expression of Cdc7-Dbf4 and ATR-HSP90-HCLK2 complex in oral cancer cell lines. The extracts of oral cancer cell lines were immunoblotted with indicated antibodies and antibody to actin as a loading control. (**B**) Clinical significance of interaction between Cdc7-Dbf4 and ATR-HSP90-HCLK2 complex in OSCC patients. The Spearman correlation coefficients were used to assess the associations between Cdc7, ATR, HCLK2, and HSP90 protein levels, which are categorized into no available, low, median, and strong according to the scores of IHC staining. (**C**) Immunohistochemical analysis of HSP90 phosphorylation at S164 in OSCC patients. Representative immunohistochemical analysis of HSP90 phosphorylation at S164 was performed by using paraffin-embedded sections of OSCC and benign oral tissue. Microscopic magnification was ×400. (**D**) Model of Cdc7-Dbf4-mediated phosphorylation events in promoting replication stress tolerance in cancer. In this study, we present the potential mechanisms that aberrant Cdc7-Dbf4 enhances DNA damage tolerance in cancer via regulating replication restart, translesion synthesis (TLS), and ATR/ATM-mediated checkpoint-HR signaling. Aberrant Cdc7-Dbf4 induces DNA lesions formation that induces replication stress and activates ATR/ATM-mediated checkpoint and HR DNA repair, and it further induces checkpoint recovery and replication restart to promote the recovery from replication stress. Cdc7-Dbf4 interacts with the ATR-HCLK2-HSP90-MRE11 complex and determines the protein stability of the complex by phosphorylating HSP90 at S164. The phosphorylation of HSP90-S164 is required for the stability and the function of ATR/ATM signaling complex cascades, which includes HR DNA repair and the recovery from replication stress. Cdc7-Dbf4 also enhances TLS and DNA damage tolerance by phosphorylating Rad18 at S434. These results illustrate that Cdc7-Dbf4 rewires different functions in DNA damage tolerance to overcome replication stress, which causes genome instability to promote tumorigenesis.

### Clinical significance of Cdc7-ATR-HCLK2 complex and the phosphorylation of HSP90-S164 in oral squamous cell carcinoma (OSCC)

We tried to link the relationship between Cdc7-Dbf4 and ATR-HSP90-HCLK2 complex in the aspect of clinical patients. Our results showed that most of oral cancer-derived cells had a higher protein level of Cdc7-Dbf4 and ATR-HSP90-HCLK2 complex than normal human oral keratinocytes (HOK) (Fig. 6A), suggesting that Cdc7-Dbf4 and ATR-HSP90-HCLK2 complex are overexpressed in Oral Squamous Cell Carcinoma (OSCC). Encouragingly, we found that the expression of Cdc7-Dbf4 corresponds with the expression of HSP90, HCLK2, TTI1, and ATR (Fig. 6A). To link their clinical significance, 154 tumor tissues from OSCC patients were used to determine the expression pattern by immunohistochemistry (IHC) analysis. The clinicopathological characteristics in 154 patients are shown in Table S4. The multivariate Cox analysis showed that cigarette smoking, differentiation, and lymph node metastasis are independent risk factors to predict poor outcome of OSCC patients (Table S5), which is similar to our recent study on OSCC^41^. Subsequently, our results indicated that the expression of Cdc7 is significantly positively correlated with HCLK2 (*P* = 0.001) and ATR (*P* = 0.037); it has the same trend with the expression of HSP90 (Fig. 6B). To further link the clinical significance of the HSP90-S164 phosphorylation in cancer tissues, 20 samples of tumor tissues and 3 benign tissues from OSCC patients were used to determine the pattern of HSP90-S164 phosphorylation by IHC analysis. The positive staining was observed in the majority of OSCC tumor tissues (18/20, 90.0%), whereas absent staining was observed in benign tissue (Fig. 6C). These results indicate that the level of Cdc7-Dbf4 is correlated with the one of ATR; the phosphorylation of HSP90 at S164 by Cdc7-Dbf4 is overexpressed and detected in OSCC patients.

## Discussion

Human Cdc7-Dbf4 remains active and functions as an upstream regulator to monitor S-checkpoint signaling in responses to replication stress^18,19^. Our previous finding suggests that increased Cdc7-Dbf4 inhibits genotoxin-induced apoptosis to enhance the survival of oral cancer cells upon DNA damage^23^. Therefore, Cdc7-Dbf4 may perform its kinase activity to phosphorylate its downstream substrates to regulate checkpoint function to enhance the survival of cancer cells under replication stress. In this study, we provided the compelling evidence that Cdc7-Dbf4 interacts with HCLK2-HSP90-Mre11 complex and phosphorylates HSP90-S164 to enhance ATR/ATM checkpoint signaling, HR DNA repair, and checkpoint recovery, which enhances replication stress tolerance for the survival of cancer cells.

We identified HSP90 as a downstream substrate of Cdc7-Dbf4 to enhance ATM/ATR-mediated checkpoint and DNA repair upon replication stress. HSP90 is a molecular chaperone with more than 200 identified client proteins^24^, including ATR^28^, Mre11/Rad50/Nbs1 complex^29^, HCLK2-TTI1/2 complex^30,42,43^, BRCA1^44^, and REV-1^45^ that are accountable for the activation of ATR signaling, DNA repair, or trans-lesion synthesis (TLS). HSP90 also acts as a facilitator of tumor progression, morphological evolution, and treatment-resistant phenotypes^26,46,47^. We found that the human Cdc7-Dbf4 kinase phosphorylates HSP90-S164 that determines the stability of HSP90 and the association with its clients in the ATR/ATM signaling. Consistently, the chaperone function of HSP90 is regulated by post-translational modification^31,32^. For example, the phosphorylation of HSP90 at Thr-22 by CK2 is essential for the binding of HSP90 with its cochaperone^48^. The phosphorylation of C-terminus HSP90 determines the choice of its binding to different co-chaperones of HSP90 that decides the folding or degradation of its client proteins^49^. However, the issue that whether the phosphorylation of HSP90 by Cdc7-Dbf4 regulates the ATR/ATM-mediated signaling through other phosphorylation sites will need further investigation.

Dysregulation in DNA replication is related to genome instability, a hallmark of cancer^50^. Cancer cells rewire DNA damage response and induce DNA repair to overcome replication stress barriers for tumor progression^51^. With its role in the initiation of DNA replication, upregulated Cdc7 largely activates the initiation, which likely accounts for the observed fork stalling (ssDNA accumulation) and subsequent forks collapse (DSB formation). We indeed observed that overexpression of Cdc7 causes the accumulation of ssDNA and DSBs that are the signals for checkpoint activation and DNA HR repair. This result is consistent with the report showing that overexpression of Cdc7 and Dbf4 does not always accelerate S phase progression but rather leads to cell-cycle arrest^52^ and with the fact that increased initiation of DNA replication leads to slowed fork progression^53^. We tried to link Cdc7 to DNA recombination and find a possible mechanism of how increased Cdc7 promotes HR DNA repair. Cdc7 has been shown to promote meiotic recombination by phosphorylation of Mer2 and Rec8 in yeast^13,54^. Recently, it has been shown that Cdc7‐Dbf4 kinase acts as a modulator of homologous recombination by regulating the Mus81‐Mms4 resolvase during mitosis in budding yeast^55^. In addition, RPA phosphorylation and Mus81-mediated formation of DSBs are required for HR-dependent replication restart and recovery of stalled forks^56^. In light of this, we proposed that overexpression of Cdc7 promotes HR may be also connected to DSBs formation and MRN complex recruitment, a DSB sensor and DNA repair component^57^, because Cdc7-Dbf4 is able to interact with MRN complex (Fig. 5). Although HR repair is often described as “error-free” and a primary pathway for stalled and collapsed fork restart^58^, the HR process in fact was shown as a highly error-prone that causes genomic instability in cancer^59–62^.

We further found that highly-upregulated Cdc7 promotes not only HR DNA repair but fork recovery, replication restart, and TLS during replication stress, which enhances DNA damage tolerance and cell survival in cancer. Consistently, substantial evidences showed that Cdc7-Dbf4 participates in the S-phase checkpoint recovery and TLS from fork arrest upon replication stress^19,20,22,63,64^. Upon removal of replication stress, Cdc7-mediated phosphorylation is required for S-phase recovery and fork stability^19,22^. It is possible that Cdc7-Dbf4 performs its kinase activity to phosphorylate its unknown substrates to regulate checkpoint function and resume replication restart and S phase progression. For instance, Cdc7-Dbf4 may facilitate checkpoint recovery and replication restart by releasing phosphorylated Rad9 from DNA damage sites in yeast^65^ and by promoting the dissociation of ATR/ATM-dependent phosphorylated Mre11 from chromatin (Fig. 5H), which suggests that MRN dissociation from DNA is involved in the checkpoint recovery following DSB repair. Taken together, we suggest that overexpression of Cdc7 not only causes more DNA lesions to activate checkpoint and HR DNA repair during the early stage of replication stress but also triggers TLS and replication restart as a compensatory mechanism for the survival of cancer cells.

Cancer cells rewire DNA damage response and induce DNA repair to overcome replication stress barriers for tumor progression, which also is a major cause of radio- or chemo-resistance^10,51^. In present study we present a novel function of excess Cdc7-Db4 in overriding replication stress to promote cancer cell survival and tumorigenesis by the phosphorylation of HSP90-S164 that enhances the ATR/ATM-mediated signaling and HR DNA repair. Cancer cells bearing checkpoint/repair-deficiency or oncogene-induced replication stress paradoxically increase ATR-Chk1 signaling expression to tolerate the stress for proliferation and survival^9,10,66^. In addition, HSP90 is often exploited by cancer cells during tumor progression to promote survival and metastasis^26,67^ by binding its oncoprotein clients, such as AKT^25^ and ATR^28^. Thus, it isn’t surprising that ATR kinase and HSP90 are selected to be promising targets of anti-cancer drug^68,69^. For example, HSP90 inhibitor, geldanamycin, 17-AAG, 17-DMAG, ganetespib, or NVP-AUY922, is currently being explored in combination therapy or clinical trials in various human malignancies^28,29,70^. However, several mechanisms of drug resistance for HSP90 inhibitor treatment were found^71,72^. Our new finding will provide valuable information for a specific type of adjuvant combination therapy that combines the inhibitors of Cdc7, HSP90, and/or ATR to block DNA synthesis and exploit defects in DNA repair/checkpoint of tumor cells^73^. Cdc7-Dbf4 kinase also is an excellent target for therapeutic strategies that not only block DNA synthesis at the beginning but also sensitize cancer cells to DNA damage by targeting HSP90/ATR/ATM, which may a promising approach to enhance the therapeutic effect of radiation or chemotherapy.

In summary, this study demonstrates that human Cdc7-Dbf4 plays multiple roles in replication stress tolerance (Fig. 6D). Aberrant Cdc7-Dbf4 induces replication stress that causes DNA lesions and activates the S-phase ATM/ATR checkpoint and HR repair. Cdc7-Dbf4 interacts and stabilizes the HCLK2-HSP90-Mre11 complex by phosphorylating HSP90 at S164, which are required for the activation of ATM/ATR signaling cascade. Cdc7-Dbf4 rewires and coordinates ATM/ATR-mediated signaling, HR DNA repair, TLS, and replication restart to promote recovery from replication stress. This study presents a novel function of Cdc7-Db4 in overriding replication stress to activate ATR/ATM signaling, and it opens a new direction to overcome replication stress tolerance in the defects in DNA repair/checkpoint in clinical by using a combination cancer therapy that combines the inhibitors of Cdc7, HSP90, and/or ATR.

## Methods

### Patients and clinical sample

A total of 154 cases of OSCC were chosen for IHC analysis based on availability of archival human oral tissue blocks from diagnostic resection specimens in the Departments of Pathology at Mackay Memorial Hospital, Taipei, Taiwan. The informed consent was obtained from each patient before the specimen was collected into the archive. The experiment was performed with approval from the Institutional Review Board (IRB number: 13MMHIS188). The main clinical characteristics of the 154 patients selected for this study are detailed in Table S1. All experiments were performed in accordance with relevant guidelines and regulations.

### Cell culture and cell treatment

U2OS, 293T, and oral cancer cell lines were cultured in medium as described previously^19,23,74^. For HU release experiment, U2OS cells infected with Ad-Cdc7 (2.4 or 7.2 × 10^8^ pfu/ml) or Ad-GFP for 24 h were synchronized with 2 mM HU for 24 hours prior to collection. The cells were collected at indicated time after HU release and were examined by FACS analysis or Western blot.

### Western blot analysis and antibodies

Western blot analysis was performed as described before^19,23^. Antibodies to human HCLK2 were generated by immunizing rabbits with using OVA-conjugated peptide (RIRSKTQRLSKGGPRQGPAG). Antibodies to human HSP90-pS164 and Rad18-pS434 were generated by immunizing rabbits with OVA-conjugated peptide DDEQYAWE[pS]SAGGS and IQEVLSS[pS]ESDS, respectively. Antibodies to human Dbf4 and Dbf4 Ser-539 phospho-specific were generated as described previously^19^. Antibodies used in this study were purchased as indicated: Antibody to Cdc7 (DCS-341) from Thermo Scientific; Rad52, cleaved Caspase-3 (Asp175, 5A1E), phospho-Chk1 (S317), phospho-Chk2 (T68), phospho-NBS1(S343), p53, and phospho-p53 (S46) antibodies from Cell Signaling Technology; Cdc6, Cdc45, cyclin A, cyclin D1, PCNA, and Ku70 from Santa Cruz Biotechnology, Inc.; ATR antibody from Abnova; phospho-ATR(T1989) antibody from MyBioSource; HSP90α and His-tag antibodies from Abcam; Mre11, RPA1, and RPA2 antibodies from Calbiochem Merck; Myc (9E10), Actin, γ-H2AX (Ser139), Rad50, Rad51 phospho-ATM (S1981), V5-Tag, phospho-serine, phospho-threonine, and phospho-Histone3 (S10) from Millipore; Phospho-MCM2 (S108), Phospho-MCM2 (S53), Phospho-MCM2 (S40/41), and Phospho-RPA2 (S4/S8) from Bethyl Laboratories; BrdU, MCM2, cyclin B1, and cyclin E from BD Biosciences; FLAG (M2) from Sigma-Aldrich; Actin, ATM, Cdt1, NBS1 and GAPDH from GenTex.

### Adenovirus construction and infection

Adenoviruses encoding GFP (Ad-Vec) and human Cdc7 (Ad-Cdc7) were generated and purified by using the AdEasy system^75^ and as described previously^19^. Large scale purification of adenovirus from 293 cells was performed by CsCl density gradient centrifugation. The concentration of purified virus was measured OD_260_ using the equation 1OD_260_ ≈ 10^12^ pfu^76^. Recombinant adenoviruses encoding Cdc7KD (D196N) was a gift from Dr. Xiaohua Wu (The Scripps Research Institute, La Jolla, CA, USA).

### Cell viability and survival assay

Cell viability assay was examined using the CellTiter 96 AQueous One Solution Cell Proliferation Assay Kit (Promega, USA) according to the manufacturer’s recommendations. Percentage of cell viability was analyzed and normalized against the untreated controls.

### Comet Assay

The neutral comet assay was performed by using the comet assay kit (4250-050-K; Trevigen, Gaithersburg, MD) according to the manufacturer’s recommendations with minor modification^19^. Briefly, U2OS were stained with SYBR Green (Trevign; 1:10000 dilution), visualized by fluorescence microscope, and analyzed. Comet tails are formed in the cells carrying DSBs. The relative length and intensity of SYBR green-stained nuclei (comets) were proportional to DNA damage in individual nuclei. This was quantified using an algorithm for Olive tail moment on the CometScore software (TriTek Corporation, Sumerduck, VA, USA). Fifty cells were counted for each experiment. Olive moment is plotted.

### Fluorescence activated cell sorting (FACS) analysi*s*

Cells were washed twice with PBS (2.7 mM KCl, 1.0 mM KH_2_PO_4_, 137 mM NaCl, 10 mM Na_2_HPO_4_, pH 7.4), trypsinized, followed by washing once with cold PBS. Cells were fixed with ice cold 70% ethanol overnight at 4 °C and stained with propidium iodide solution which containing 2 mM MgCl_2_, 1% Triton X-100, 100 ng/ml RNase A and 0.1 mg/ml propidium iodide (Sigma-Aldrich, St Louis, MO, USA). The labeled cells were analyzed with a FACSCalibur flow cytometer (Becton-Dickinson Biosciences, Mansfield, MA, USA) using CELLQUEST software (Becton-Dickinson).

### Immunofluorescence staining

The immunofluorescence staining was determined as described previously^77^ with minor modifications. U2OS cells cultured in glass slides were infected with Ad-Myc-Cdc7 (1.2 × 10^8^ pfu) or Ad-Vec for 48 h. The cells were fixed in 3.7% formaldehyde in PBS for 10 min at room temperature and permeabilized with 0.1% Triton X-100 for 5 min. After blocked in 3% BSA, the cells were incubated with primary antibody 1 h at 37 °C and then with the anti-mouse fluorescence-labeled secondary antibody 1 h at room temperature. DNA was stained with 4,6-diamidino-2-phenylindole dilactate (DAPI, Invitrogen). For 5-Bromo-2′-deoxy-uridine (BrdU) incorporation assay, U2OS cells cultured in glass slides were infected with Ad-Myc-Cdc7 (1.2 × 10^8^ pfu/ml) or Ad-Vec for 48 h. U2OS cells infected with Ad-Myc-Cdc7 or Ad-Vec were treated with 30 μM BrdU (Sigma) for 15 min, which could be incorporated into DNA during the S phase, and the cells were fixed with 3.7% paraformaldehyde. Subsequently, the cells were incubated with 2 M HCl for 30 min at 37 °C to denature DNA and treated with 0.1 M borate buffer (Na_2_B_4_O_7_, pH 8.5) for 5 min to neutralize the acid. After 3% BSA blocking for 1 h at room temperature, the cells were incubated with mouse anti-BrdU antibody (1:200) or anti-γ-H2AX (1:200) 1 h at 37 °C, then with goat anti-mouse DyLight^TM^ fluorescence-conjugated secondary antibody for 1 hour at RT. The above experimental results were analyzed by an Olympus BX51 fluorescence microscopy.

### Homologous recombination (HR) assay

HR assay was performed as described previously^78^ with minor modification. U2OS cells transfected with DR-GFP were used for HR assay (a generous gift from Dr. Maria Jasin)^35^. To examine HR repair, we performed the DSB repair assay using I-SceI endonuclease to introduce genomic DSBs in U2OS cells which are transfected by DR-GFP reporter containing two mutated GFP cassettes, SceGFP and iGFP. To determine the effect of Cdc7 overexpression on efficiency of HR-mediated DSB repair, U2OS (DR-GFP) cells were transfected with I-Sce1 vector only, or co-transfected with pcDNA3-myc-Cdc7 or pcDNA3-myc-Cdc7KD using PolyJet *in vitro* DNA transfection reagent (SignaGen laboratories). U2OS cells were transfected with eGFP as a positive control, while U2OS (DR-GFP) cells alone were as a negative control. 24 h after transfection, U2OS (DR-GFP) cells were selected positively with puromycin (2 μg/ml) and the cells were incubated for 24 h. Subsequently, flow cytometric analysis was used to determine the percentage of GFP-positive cells.

### DNA replication foci analysis

DNA replication foci were visualized by incorporation of chlorodeoxyuridine (CldU) and iododeoxyuridine (IdU) into DNA. U2OS cells were labeled with 100 μM CldU (Sigma Chemical Co., St. Louis, MO) or 20 μM IdU (Sigma Chemical Co., St. Louis, MO) for different time intervals. Primary antibodies for CldU (rat anti-BrdU (1:100); BD Biosciences) and IdU (mouse anti-BrdU (1:100); ABcam) were diluted in 3% BSA and incubated at 37 °C for 1 h. The slides were incubated with secondary antibodies (CldU, donkey anti-rat Alexa Fluor 488 [Molecular Probes/Invitrogen]; IdU, goat anti-mouse 594 [Jackson Immunoresearch]) for 1 h. Images were visualized by using a Leica confocal microscope.

### *In vitro* Kinase Assay

*In vitro* kinase assay was performed using 293T cells that is transiently transfected with FLAG-tagged Dbf4 and Cdc7 plasmid or using human GST-tagged Cdc7-Dbf4 kinase complex purchased from SiganalChem. Anti-FLAG immunoprecipitates or GST-tagged Cdc7-Dbf4 kinase were incubated with the purified His-HSP90α WT, deletion mutants, or GST-MCM2 (aa1-169) as a positive control in Cdc7 kinase buffer (25 mM HEPES pH 7.5, 50 mM NaCl, 10 mM MgCl_2_, 1 mM DTT, 10 μM ATP) and in the presence of 10 μCi [γ-^32^P]ATP and phosphatase inhibitors (10 mM NaF, 50 mM β-glycerophosphate) at 30 °C for 40 mins, followed by the addition of SDS-PAGE sample buffer to stop reaction. Phosphorylated radioactive proteins were separated by SDS-PAGE and detected by autoradiography of the dried gels.

### Quantitative reverse transcription-polymerase chain reaction (qRT-PCR)

The qRT-PCR was performed as described previously^23^. The primer sequences used are listed below. Cdc7-F: 5′- CAA AGT GCC CCA ATC AAA CT-3′, Cdc7-R: 5′-TGGGCCAAAGCA GTTAAATC-3′; β-actin-F: 5′-CTCTTCCAGCCTTCCTTCCT-3′, β-actin-R: 5′-AGC ACT GTGTTGGCGTACAG-3′; HSP90α-F: 5′-ATGAAACTGCGCTCCTGTCT; HSP90α- R: TTC TTCCATGCGTGATGTGT. All amplifications were performed in triplicate.

### Immunohistochemistry (IHC) staining

IHC analysis was performed on an automatic staining machine (BenchMark XT, Ventana Medical Systems, Tucson, AZ, USA) using the iVIEW 3, 3-diaminobenzidine (DAB) detection kit (Ventana Medical Systems). Paraffin sections (4 μm) containing human of the 110 OSCC tissues were routinely deparaffinized, hydrated, and heated to 95~100 ^o^C for 4 min to induce antigen retrieval. After inactive the endogenous peroxidase activity, IHC staining was performed with anti-Cdc7 (Thermo Scientific DCS-341, 1:20). All sections were finally incubated with iVIEW copper for 4 min to enhance the signal intensity, then counterstained with hematoxylin, dehydrated, mounted, and observed by a Nikon Eclipse E600 light microscope (Tokyo, Japan). Pictures were acquired using Tissue-Faxs software (TissueGnostics). The staining intensity was estimated in a 4-scored scale (0, Negative staining; 1+, weak; 2+, moderate; 3+, strong intensity). The fraction of stained cells was scored according to the following criteria: Score 0 (no stained or <10% stained cells), Score 1 (11–50% stained cells), Score 2 (51–80% stained cells), Score 3 (>80% stained cells).

### Statistical analysis

Multivariate Cox proportional hazard model was used to estimate hazard ratios with adjustments for age and gender. We considered a statistical significance if a *P*-value was < 0.05. All data was analyzed using the R statistical software (version 3.1.1). Parametric Student’s t test was used to judge the significance of difference between conditions of interest. The association between Cdc7 protein level (scoring of the IHC staining) and the quantified clinicopathological features of tumors were examined by χ2 test. Cox proportional hazard regression model and log-rank test was applied for analysis of survival data. In all analysis, a P value of < 0.05 was considered as statistically significant (Student’s t test, *p < 0.05, **p < 0.01, and ***p < 0.001).

## Electronic supplementary material

Supplementary Information

Table S1-S3
