# Supplementary material for: Cdc7-Dbf4-mediated phosphorylation of HSP90-S164 stabilizes HSP90-HCLK2-MRN complex to enhance ATR/ATM signaling that overcomes replication stress in cancer
Source: Sci Rep. 2017 Dec 5;7:17024. doi: 10.1038/s41598-017-17126-2 (PMC5717001; doi:10.1038/s41598-017-17126-2)
Supplement: Supplementary file 1 — Supplementary Information [file 41598_2017_17126_MOESM1_ESM.docx]

**Cdc7-Dbf4-mediated phosphorylation of HSP90-S164 stabilizes HSP90-HCLK2-MRN complex to enhance ATR/ATM signaling that overcomes replication stress in cancer**

An Ning Cheng^1,2^#, Chi-Chen Fan^3,4^#, Yu-Kang Lo^1^, Cheng-Liang Kuo^1^, Hui-Chun Wang^5^, I-Hsin Lien^1^, Shu-Yu Lin^6^, Chung-Hsing Chen^1^, Shih Sheng Jiang^1^, I-Shou Chang^1^, Hsueh-Fen Juan^7,8^, Ping-Chiang Lyu^2^, and Alan Yueh-Luen Lee^1,9^*

**Supplementary materials and methods**

**Cell culture and cell treatment**

U2OS and 293T cells were cultured in medium containing Dulbecco’s modified Eagle’s essential medium (DMEM), supplemented with 5% fetal bovine serum (FBS) and 5% super calf serum. All medium contain penicillin 100 Units/mL, streptomycin 100 μg/mL except mentioned. Oral cancer cell lines, HSC-3, and FADU were grown in a culture medium containing DMEM, supplemented with 5% heat-inactivated FBS and 5% super calf serum. SCC-9 and SCC-15 cells were cultured in medium containing a 1:1 mixture of DMEM/F12 medium, supplemented with 10% FBS. OC3 cells were cultured in medium containing a 1:1 mixture of DMEM/K-SFM medium, supplemented with 10% FBS and OEC-M1 cells were cultured in medium containing RPMI 1640 medium, supplemented with 10% FBS. Oral pre-cancer lesion cells DOK (dysplastic oral keratinocyte) were cultured in medium containing DMEM, supplemented with 10% FBS ([Chang et al., 1992](#_ENREF_15)).

**Whole cell lysate and chromatin isolation**

The cell lysate preparation and chromatin isolation was performed as described previously ([Lee et al., 2012](#_ENREF_54)). Cells were lysed in NETN (150 mM NaCl, 1 mM EDTA, 20 mM Tris-Cl pH 8.0, 0.5% NP-40 (v/v)) containing protease and phosphatase inhibitors (50 mM sodium fluoride, NaF, 0.1 mM sodium orthovanadate, NaVO_4_). For chromatin isolation, cells were washed with phosphate-buffered saline (PBS), collected, and resuspended in CSK buffer (10 mM HEPES pH 6.8, 100 mM NaCl, 300 mM sucrose, 3 mM MgCl_2_, 1 mM EGTA, 0.1% Triton X-100, 1mM DTT, and protease and phosphatase inhibitors), and incubated on ice for 10 min. Cytoplasmic proteins were separated from nuclei by low speed centrifugation at 1300 *g* for 5 min. Cytoplasmic fraction could be further centrifuged at 14400 g for 15 min to obtain Triton-soluble fraction. Isolated nuclei were washed once, incubated on ice for 5 min and lysed in CSK buffer. After centrifugation at 1,700 g for 5 min, Triton-insoluble chromatin-containing fractions were resuspended in CSK buffer and mixed with 2X SDS loading buffer. Samples were boiled for 15 min and votexed for 2 min.

**Cell viability/survival assay**

Cell viability assay was examined using the CellTiter 96 Aqueous (CTAQ) One Solution Cell Proliferation Assay Kit (Promega, USA) according to the manufacturer’s recommendations. The assay kit is a colorimetric method for determining the number of viable cells in proliferation or cytotoxicity assays, which contains a novel tetrazolium compound [3-(4,5-dimethylthiazol-2-yl)-5-(3-carboxymethoxyphenyl)-2-(4-sulfophenyl)-2H-tetrazolium, inner salt; MTS] and an electron coupling reagent (phenazine ethosulfate; PES). Briefly, cells infected with Ad-Cdc7 or Ad-Vec were incubated overnight, then the trypsinized cells (5 × 10^3^ cells/well) were seeded in 96-well plates in triplicate, followed by the treatment of hydroxyurea (HU, 5 mM, 24 h). After 24 h incubation, 20 μl CTAQ reagents per 100 μl culture medium were added and the samples were measured on the value of OD_490_ with a microplate reader (Molecular Devices, Sunnyvale, CA, USA). Percentage of cell viability was analyzed and normalized against the untreated controls.

**Shotgun proteomic identifications**

NanoLC−nanoESI-MS/MS analysis was performed on a nanoAcquity system (Waters, Milford, MA) connected to the Orbitrap Elite hybrid mass spectrometer (Thermo Electron, Bremen, Germany) equipped with a PicoView nanospray interface (New Objective, Woburn, MA). Peptide mixtures were loaded onto a 75 μm ID, 25 cm length C18 BEH column (Waters, Milford, MA) packed with 1.7 μm particles with a pore with of 130 Å and were separated using a segmented gradient in 60 min from 5% to 40% solvent B (acetonitrile with 0.1% formic acid) at a flow rate of 300 nL/min and a column temperature of 35°C. Solvent A was 0.1% formic acid in water. The mass spectrometer was operated in the data-dependent mode. Briefly, survey full scan MS spectra were acquired in the orbitrap (m/z 350–1600) with the resolution set to 120K at m/z 400 and automatic gain control (AGC) target at 106. The 15 most intense ions were sequentially isolated for HCD MS/MS fragmentation and detection in the orbitrap with previously selected ions dynamically excluded for 60 s. For MS/MS, we used a resolution of 15000, an isolation window of 2 m/z and a target value of 50000 ions, with maximum accumulation times of 200 ms. Fragmentation was performed with normalized collision energy of 30% and an activation time of 0.1 ms. Ions with singly and unrecognized charge state were also excluded. The MS and MS/MS raw data were processed by Proteome Discoverer (v 1.4.0.288; Thermo Scientific, Waltham, MA, USA) and searched against Swiss-Prot protein sequence database with the Mascot Daemon 2.4.0 server. Search criteria used were trypsin digestion, variable modifications set as carbamidomethyl (C), oxidation (M) and phosphorylation (STY) allowing up to 2 missed cleavage, mass accuracy of 10 ppm for the parent ion and 0.05 Da for the fragment ions. Phosphorylation sites and peptide sequence assignments contained in MASCOT search results were validated by manual confirmation from raw MS/MS data.

**Phosphoproteome analysis**

Proteins were extracted with 12 mM sodium deoxycholate (Sigma-Aldrich, St Louis, MO, USA), 12 mM sodium lauroyl sarcosine, 50 mM triethylammonium bicarbonate (Sigma-Aldrich), protease cocktail (Sigma-Aldrich), and phosphatase inhibitor cocktail (Sigma-Aldrich). Cell lysates were reduced with 10 mM dithiothreitol (BioShop, Burlington, Canada) at room temperature for 30 min, and carbidomethylated with 55 mM iodoacetamide (Sigma-Aldrich) in the dark at room temperature for 30 min. Alkylated proteins were digested with Lys-C (1:100 w/w; WAKO) for 2 hr and then digested with sequencing-grade modified trypsin (1:100 w/w; Promega, Mannheim, Germany) overnight. For detergent removal, the peptide mixture was combined with an equal volume of ethyl acetate (Sigma-Aldrich) and then acidified with trifluoroacetic acid (Sigma-Aldrich) to a pH of <3. The acidified sample was shaken for 1 min and then centrifuged at 15700 × g for 2 min to separate the aqueous and organic phases. The collected aqueous sample without detergents was desalted by using SDB-XC StageTips (3M, Neuss, Germany).

Tryptic peptide samples (150 μg) were dried under vacuum and then redissolved in 0.1 M TEAB solution. The control and drug-treated peptide samples were first mixed with 6 μL of 4% formaldehyde-H2 (Sigma-Aldrich) and 4% formaldehyde-D2 (Sigma-Aldrich), respectively, and then immediately 6 μL of freshly prepared 0.6 M sodium cyanoborohydride (Sigma-Aldrich) was added to each mixture. Each mixture was vigorously mixed and then reaction was allowed to proceed for 60 min at room temperature. Ammonium hydroxide (1%, 24 μL) was added to stop the reaction by reacting with the excess formaldehyde. Formic acid (10%, 30 μL) was further added with functions of ending the labeling reaction and acidifying the samples. Finally, the H-and D-labeled samples were combined at1:1 ratio and then desalted by using SDB-XC StageTips. The phosphopeptide was enriched by hydroxy acid-modified metal oxide chromatography (HAMMOC). Briefly, custom-made MOC tips were prepared by packing 0.5 mg of TiO2 beads (GL Sciences, Tokyo, Japan) into 10µL C8 StageTips. Prior to sample loading, MOC tips were equilibrated with solution A (0.1% TFA, 80% acetonitrile, and 300 mg/mL lactic acid). About 200 µg of desalted peptide mixture was mixed with an equal volume of solution A. The resulting mixture was loaded onto the MOC tips (100 µg mixed peptides per tip) and then washed with solution A and with solution B (0.1% TFA and 80% acetonitrile). Phosphopeptides were eluted with 0.5% and 5% solutions of piperidine (Sigma-Aldrich). The eluate was acidified with TFA, desalted with SDB-XC StageTips, and then vacuum-dried. The phosphopeptides were then re-suspended in 0.5% TFA and analyzed by nanoLC–MS/MS.

**Data analysis for phosphoproteome experiment**

Raw MS spectra were processed for peak detection and quantitation by using MaxQuant software version 1.3.0.5. Peptide identification was performed by using the Andromeda search engine and the Swiss-Prot database (release 87, subset human, 20265 protein entries). Search criteria used in this study were trypsin specificity, fixed modification of carbamidomethyl, variable modifications of oxidation and phosphorylation, and allowed for up to two missed cleavages. A minimum of six amino acids in the peptide length was required. The precursor mass tolerance was 3 ppm and the fragment ion tolerance was 0.5 Da. By usinga decoy database strategy, peptide identification was accepted based on the posterior error probability with a false discovery rate of 1%. Precursor intensities of already identified peptides were further searched and recalculated by using the “match between runs” option in MaxQuant. For single peptide with multiple hits, all matched proteins were counted separately. The ratio of phosphosite was normalized by MaxQuant. Phosphosites that displayed a minimum of threefold change in abundance for at least three time points in the temporal phosphoproteomic study were considered as regulated phosphosites.

**Supplementary Data**

**Supplementary Table 4.** Clinicopathological characteristics in OSCC patients (*n=*154)

| Variables | | Number of patients (%) | |
| --- | --- | --- | --- |
| Age (years old) | |  |  |
|  | $\leq$ 58 | 84 | (54.5%) |
|  | $>$ 58 | 70 | (45.5%) |
| Gender | |  |  |
|  | Female | 8 | (5.2%) |
|  | Male | 146 | (94.8%) |
| Alcohol drinking | |  |  |
|  | No | 47 | (30.5%) |
|  | Yes | 107 | (69.5%) |
| Betel quid chewing | |  |  |
|  | No | 112 | (72.7%) |
|  | Yes | 42 | (27.3%) |
| Cigarette smoking | |  |  |
|  | No | 59 | (38.3%) |
|  | Yes | 95 | (61.7%) |
| Differentiation | |  |  |
|  | Well / Moderate | 142 | (92.2%) |
|  | Poor | 11 | (7.1%) |
|  | No information | 1 | (0.6%) |
| Tumor Size | |  |  |
|  | $\leq$ 2cm | 55 | (35.7%) |
|  | $>$ 2cm | 99 | (64.3%) |
| Lymph node metastasis | |  |  |
|  | No | 106 | (68.8%) |
|  | Yes | 47 | (30.5%) |
|  | No information | 1 | (0.6%) |
| TNM stage | |  |  |
|  | In situ | 2 | (1.3%) |
|  | I, II | 31 | (20.1%) |
|  | III, IV | 116 | (75.3%) |
|  | No information | 5 | (3.2%) |

**Supplementary Table 5.** Multivariate analysis of overall survival of OSCC patients (*n=*147).

| Variables | Hazard ratio | 95% CI* | P-value |
| --- | --- | --- | --- |
| Age (years old) | 1.013 | (0.987,1.039) | 0.347 |
| Gender | 0.989 | (0.226,4.331) | 0.989 |
| Alcohol drinking | 1.046 | (0.565,1.936) | 0.886 |
| Betel quid chewing | 0.601 | (0.291,1.242) | 0.169 |
| Cigarette smoking | 1.922 | (0.980,3.768) | 0.057 |
| Differentiation (Well / Moderate vs. Poor) | 3.941 | (1.754,8.854) | 0.001 |
| Tumor Size ($\leq$ 2cm vs.$>$ 2cm) | 1.656 | (0.864,3.174) | 0.129 |
| Lymph node metastasis | 2.149 | (1.139,4.053) | 0.018 |
| TNM stage ($\leq\text{II}$vs.$>\text{II}$) | 1.289 | (0.556,2.988) | 0.554 |

*Hazard ratios with 95% confidence intervals (95% CI) were estimated

**
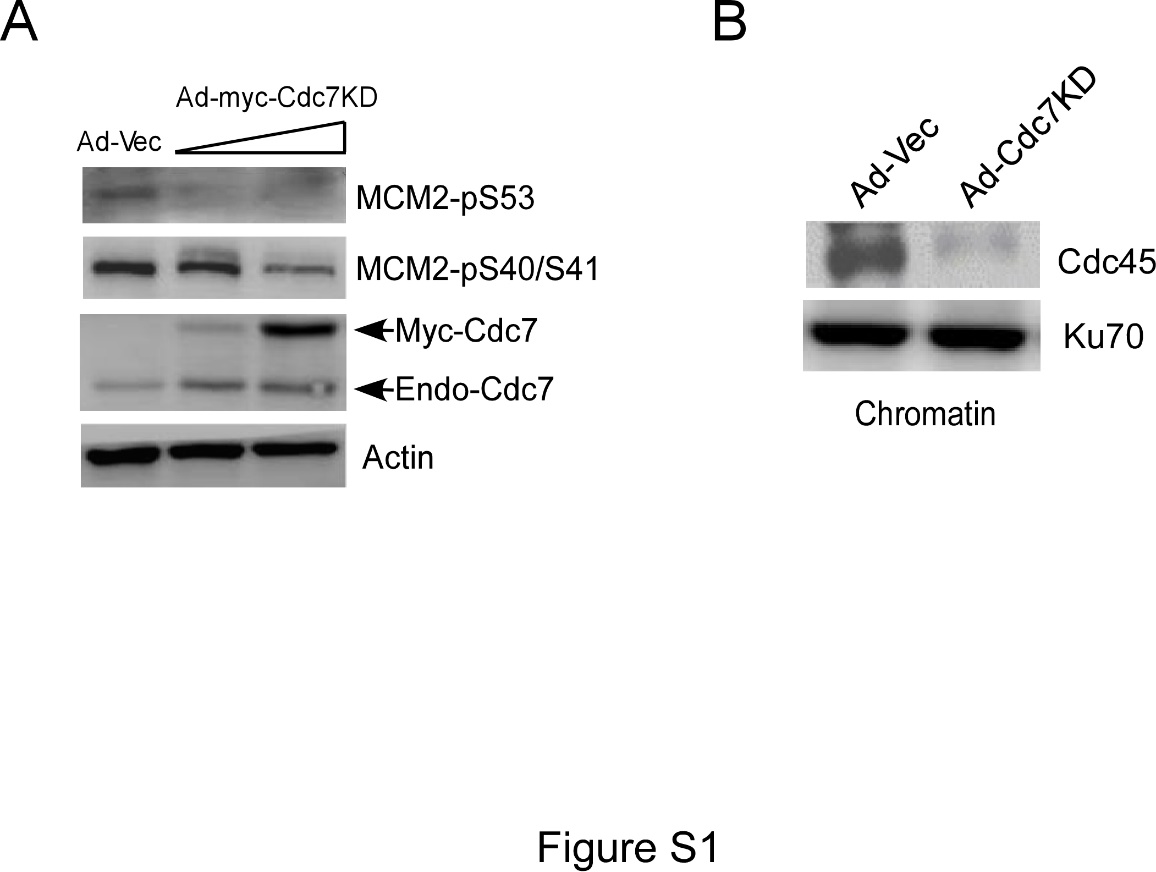
**

**Figure S1. Cdc7 activity is required for the initiation of DNA replication.**

U2OS was infected with Ad-Cdc7KD (1.2 or 3.6 × 10^8^ pfu/ml) or Ad-Vec (3.6 × 10^8^ pfu/ml) for 48h. The lysates were analyzed by Western blotting using the indicated antibodies. The antibodies to actin and KU70 were used as a loading control. The expression of endogenous Cdc7 (Endo-Cdc7) and adenovirally introduced Myc tagged Cdc7 kinase dead mutant (Myc-Cdc7KD) is shown as indicated.

**
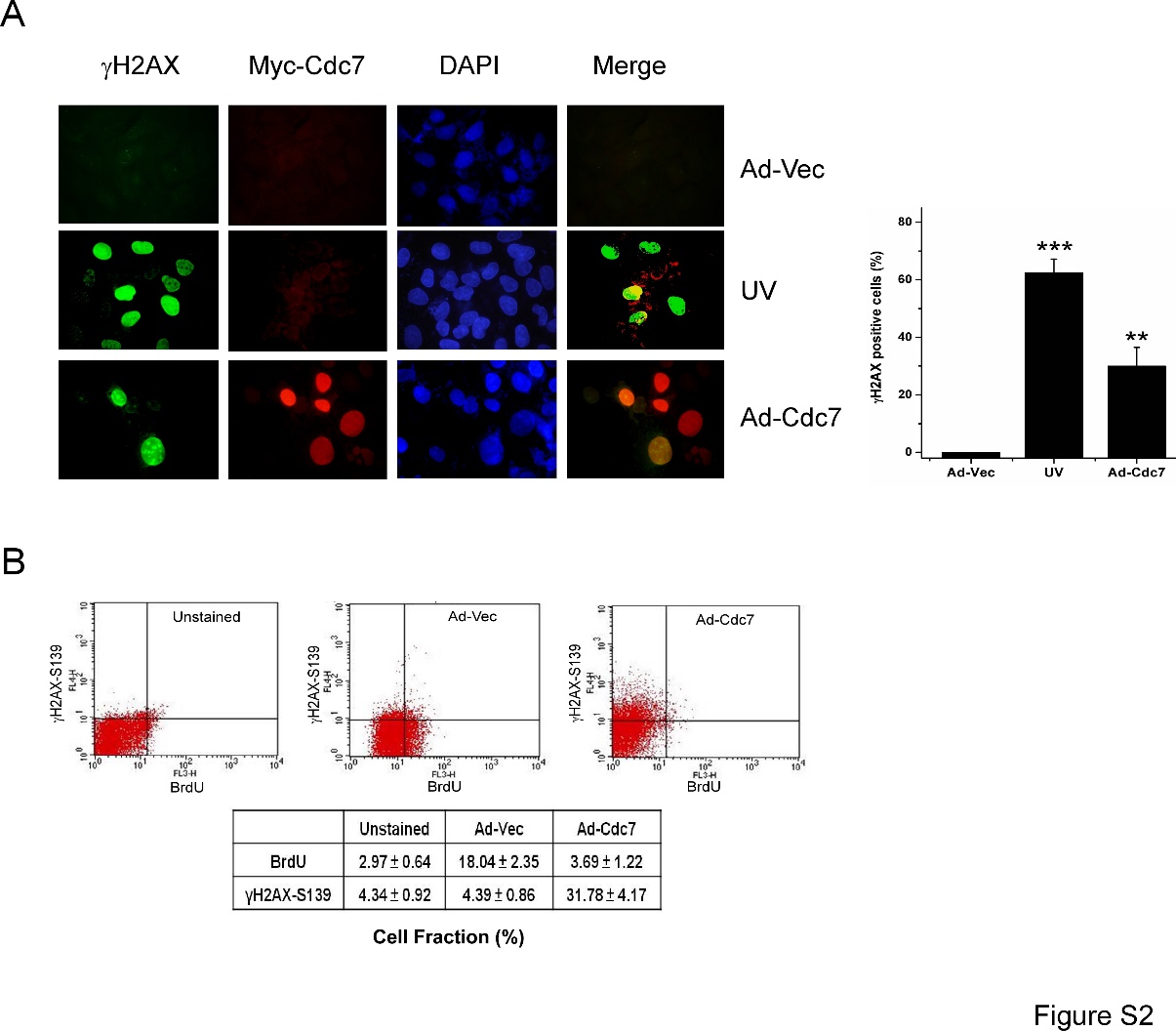
**

**Figure S2. Cdc7 overexpression increase formation of DSBs and reduces replication initiation.**

**A.** U2OS cells infected with Ad-Cdc7 (2.4 × 10^8^ pfu/ml) were treated with or without UV irradiation (50 J/m^2^) after 48h infection and immunostaining was performed by using antibodies toward γH2AX and Myc. U2OS cells treated with UV only were used as a positive control. DAPI was used for nuclear staining. The graph with error bars indicating the standard error from the average is shown in the bottom. Results are presented as range of three independent experiments. *: P <0.05, **: P <0.01, ***: P <0.001.

B. Cdc7 overexpression increases γH2AX staining and inhibits BrdU incorporation.

U2OS cells were infected with Ad-Cdc7 (2.4 × 10^8^ pfu/ml) or Ad-Vec (2.4 × 10^8^ pfu/ml). After 48h infection and BrdU incorporation for 1h, FACS analysis was performed by double staining using antibodies toward BrdU and γH2AX. The cell fraction of the cells with the standard error from the average is shown in the bottom. Results are presented as range of three independent experiments.

**
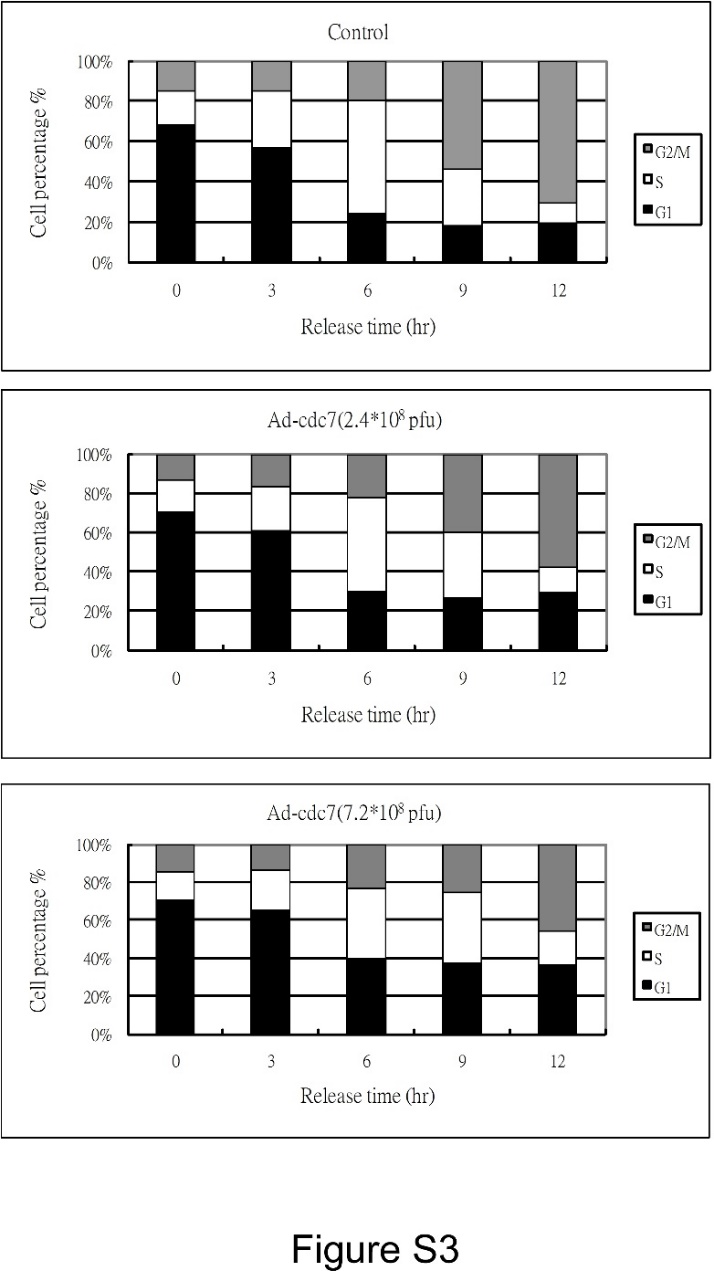
**

**Figure S3. Cdc7 overexpression delays cell cycle progression after HU treatment**

U2OS cells infected with Ad-Cdc7 (2.4 or 7.2 ×10^8^ pfu/ml) or Ad-GFP (Control, 7.2 × 10^8^ pfu/ml) for 24 hours were treated with 2 mM HU for another 24 hours and collected at indicated time after treatment. FACS analysis was performed and the percentage of the cells in G1/S, S and G2/M phase are shown in different bars. Results are presented as the average of three independent experiments.


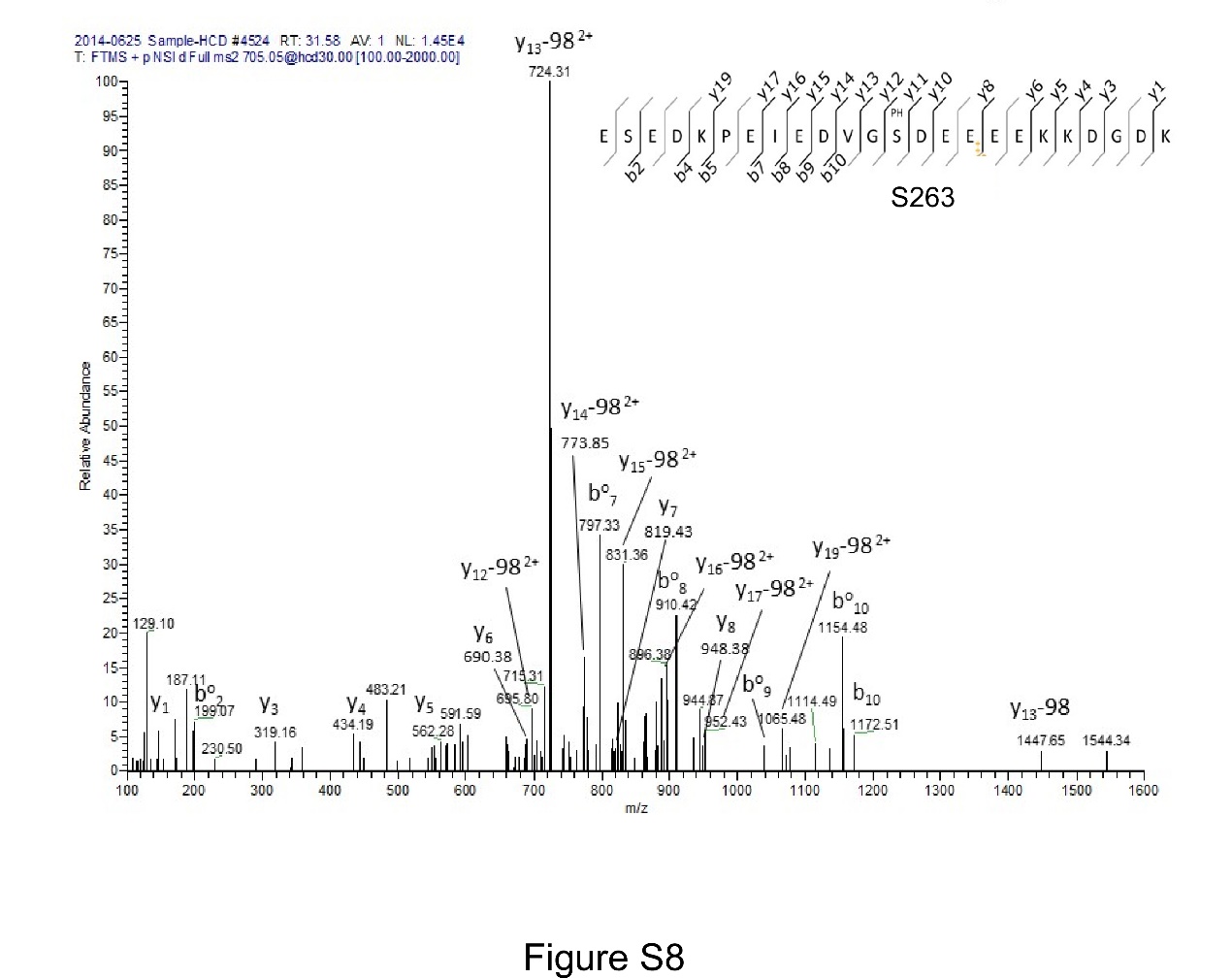


**Figure S4. Cdc7-Dbf4 phosphorylates HSP90α at Ser 263.**

Purified Cdc7-Dbf4 and HSP90α were incubated at 30⁰C for 40 mins, then the mixture was collected after in-gel digestion and used in MS/MS spectrum analysis. NanoLC−nanoESI-MS/MS analysis was performed on a nanoAcquity system. MS/MS spectrum shows [M + 2H]^2+^ (m/z 705.05) ion of the peptide ESEDKPEIEDVG[pS]DEEEEKKDGDK from the HSP90α protein, which is a phosphorylated peptide. The finding of product ion y11 that carries a phosphate indicates that Ser263 was phosphorylated. Residues bearing phosphate moieties are indicated with p. “b” and “y” ion series represent fragment ions containing the N- and C-termini of the peptide, respectively.


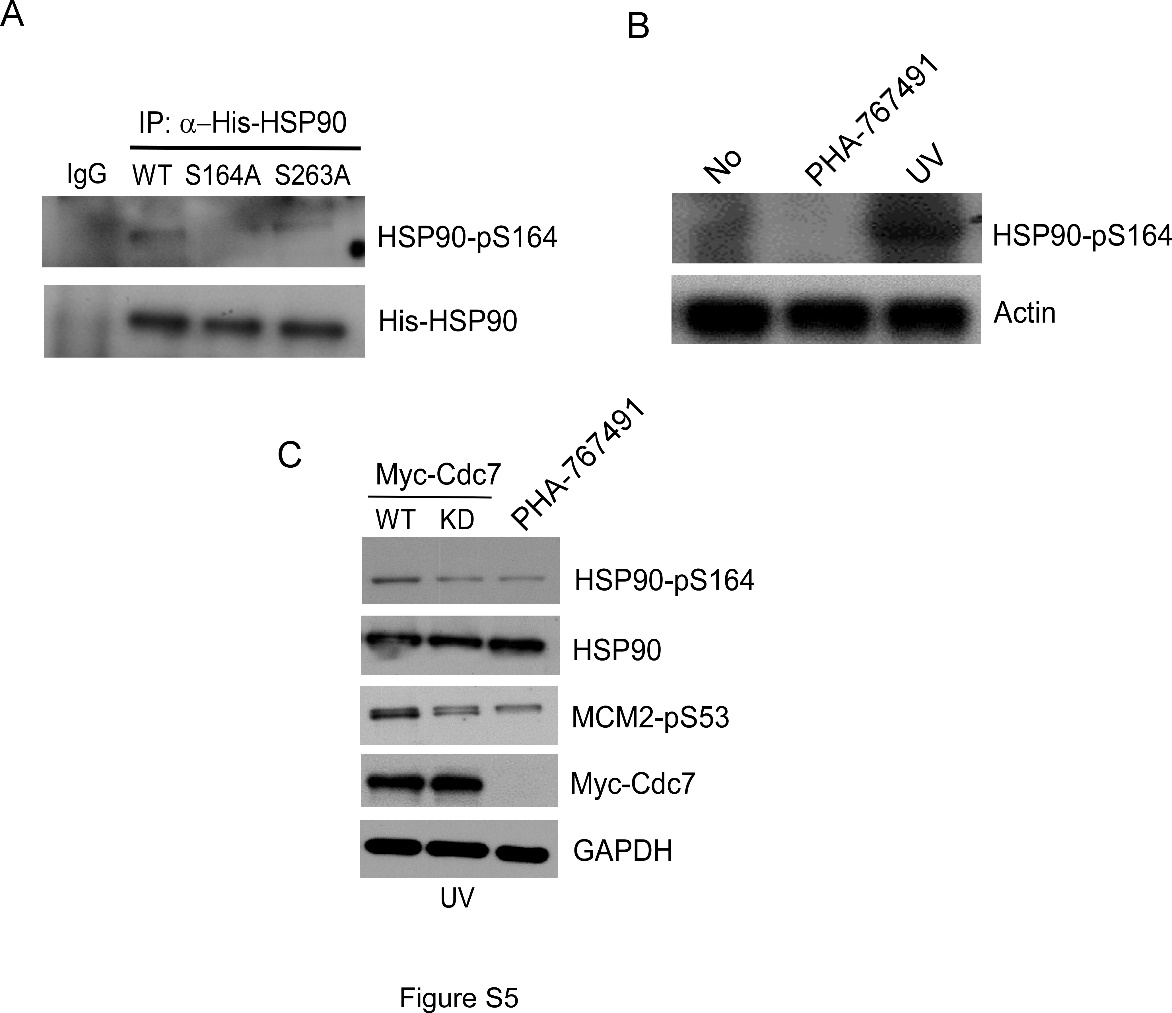


**Figure S5.** **Validation of the specificity of purified phospho-specific antibody to HSP90α Ser164.**

**A.** HSP90 phospho-mutant at S164 or S263 was generated. 293T cells were transfected with plasmids encoding His-tagged HSP90 S164A or S263A followed by co-immunoprecipitation with anti-His antibody. Immunoblotting was performed as indicated.

**B.** U2OS cells were treated with PHA-767491 (5μM for 3h ) or UV radiation (50 J/m^2^ , recovery for 2 h). The lysates were analyzed by immunoblot using the indicated antibodies.

**C.** U2OS cells were transfected with plasmids encoding Myc-Cdc7 or Myc-Cdc7 KD followed by treatment with UV radiation (50 J/m^2^, recovery for 2 h). The treatment with PHA-767491 (5 μM for 3h) was used as a positive control experiment. The lysates were analyzed by immunoblot using the indicated antibodies.

**
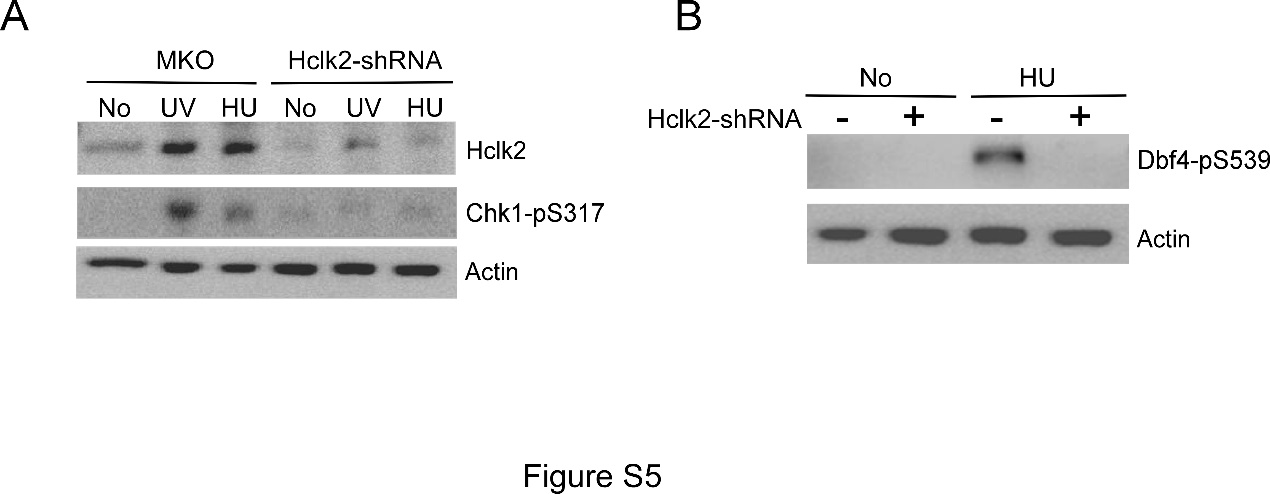
**

**Figure S6. HCLK2 is required for activation of ATR-Chk1 pathway.** U2OS cells transfected with HCLK2-shRNAs were treated with or without UV(50 J/m^2^, 3h after) or HU (1 mM, 16 h). The lysates were analyzed by immunoblot using the indicated antibodies. The same blot was used to detect Actin as a control of loading.


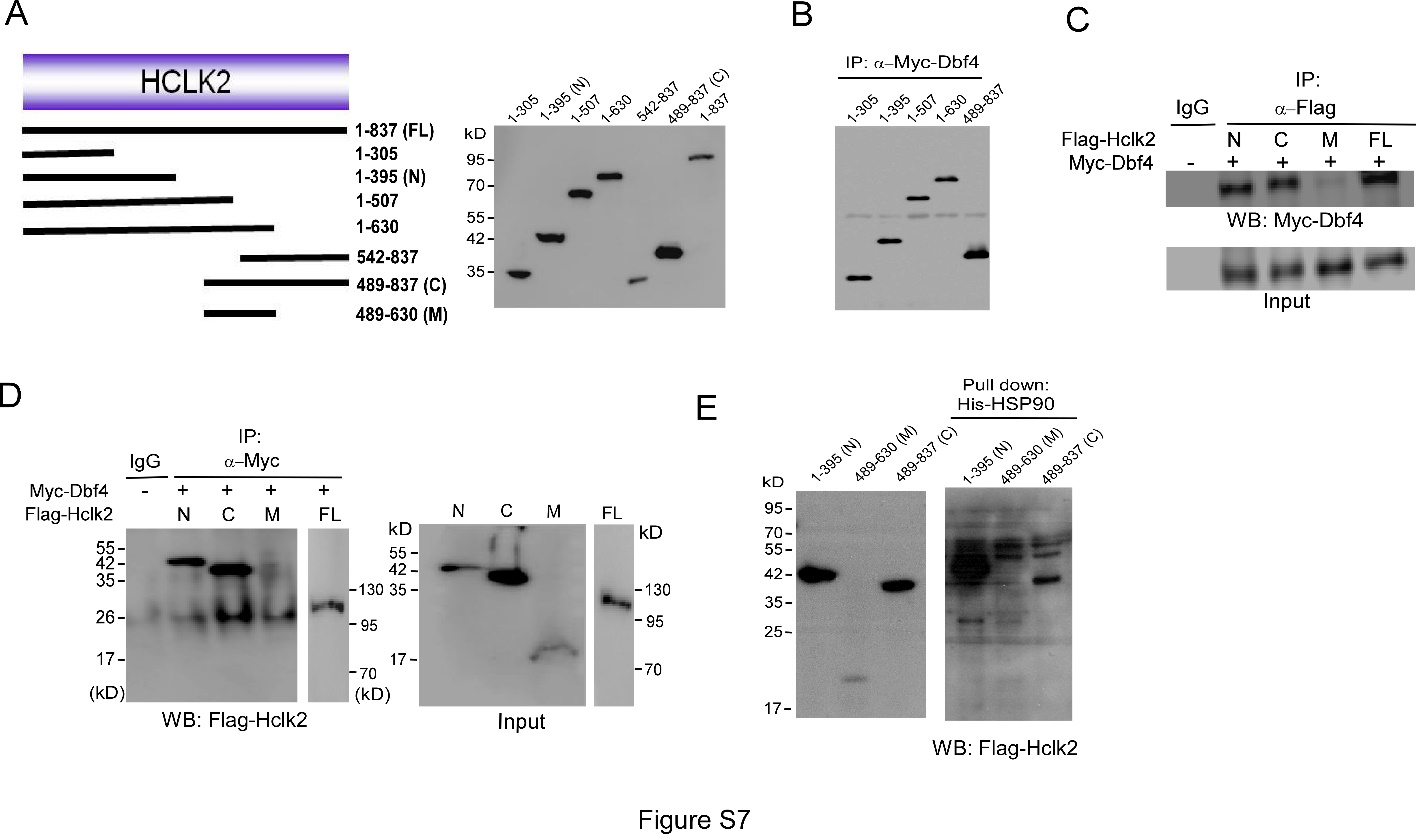


**Figure S7. Mapping of the fragments of** **HCLK2 required for interaction with Cdc7-Dbf4 and HSP90.**

**A. Different fragments of HCLK2 were showed in the scheme.**

Expression of HCLK2 fragments is shown by Western blotting (right).

**B, C and D. HCLK2 interacts with Cdc7-Dbf4 through N-terminal (aa1-488) and C-terminal (aa631-837) domain.**293T cells were transfected with plasmids encoding Flag-tagged HCLK2 fragments and Myc-Dbf4. Then the collected cell lysate was used to pull-down the tagged proteins with Flag-or Myc-resin. After 3 hours binding, the resin was washed and prepared for immunoblotting.

**E. HCLK2 interacts with HSP90 through N-terminal (aa1-395) and C-terminal (aa631-837) domain.**

293T cells were transfected with plasmids encoding Flag-tagged HCLK2 fragments and His-tagged HSP90α. Then the collected cell lysate was used to pull-down the His-tagged HSP90α. After 3 hours binding, the resin was washed and prepared for immunoblotting.


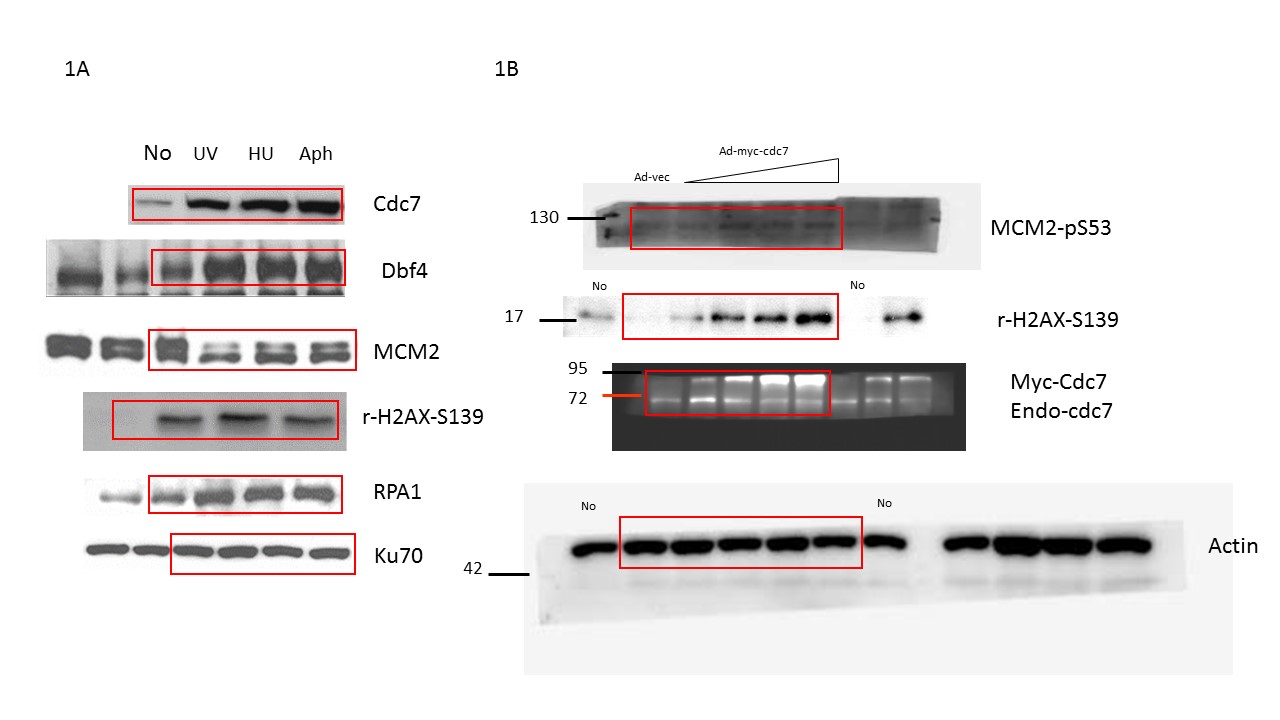


**
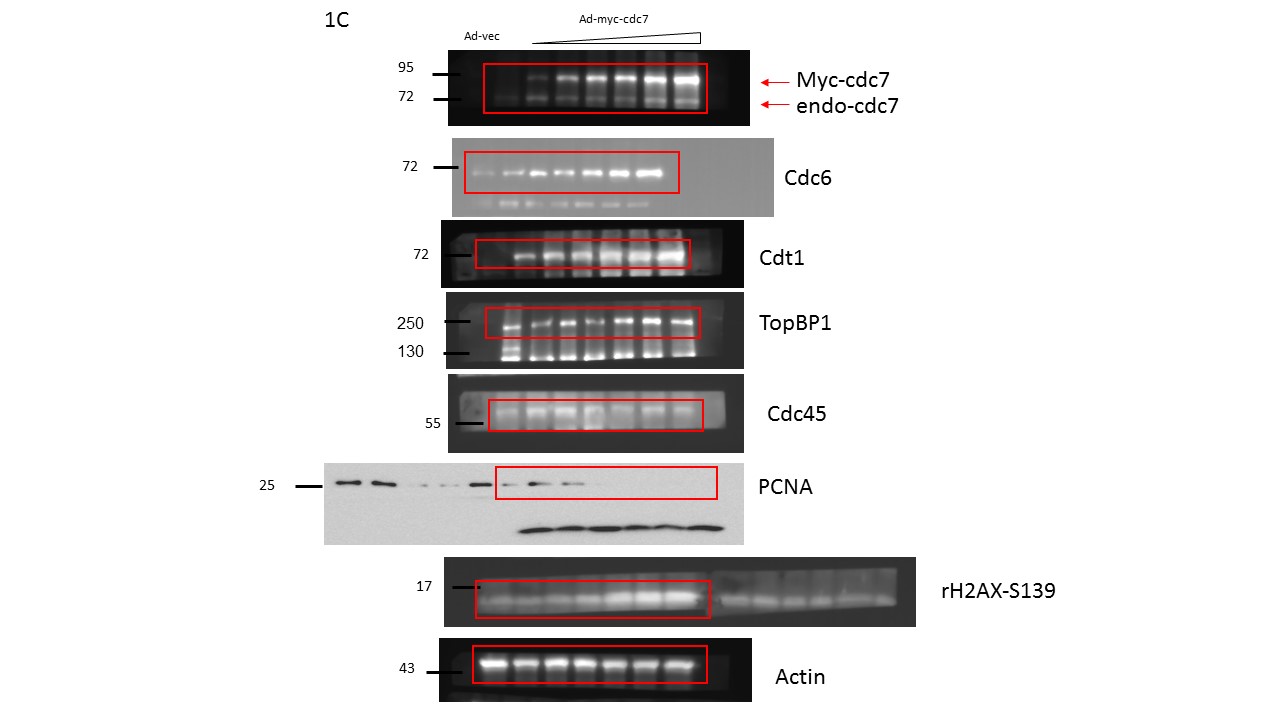
**

**
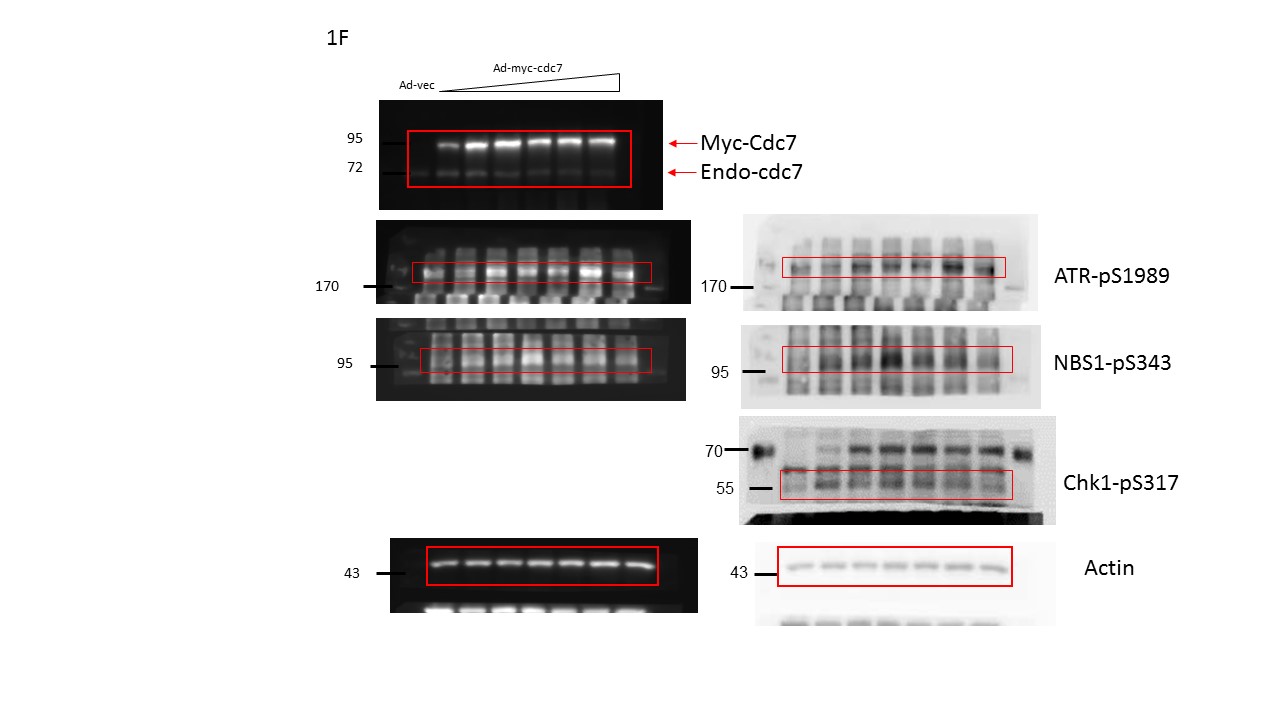
**

**
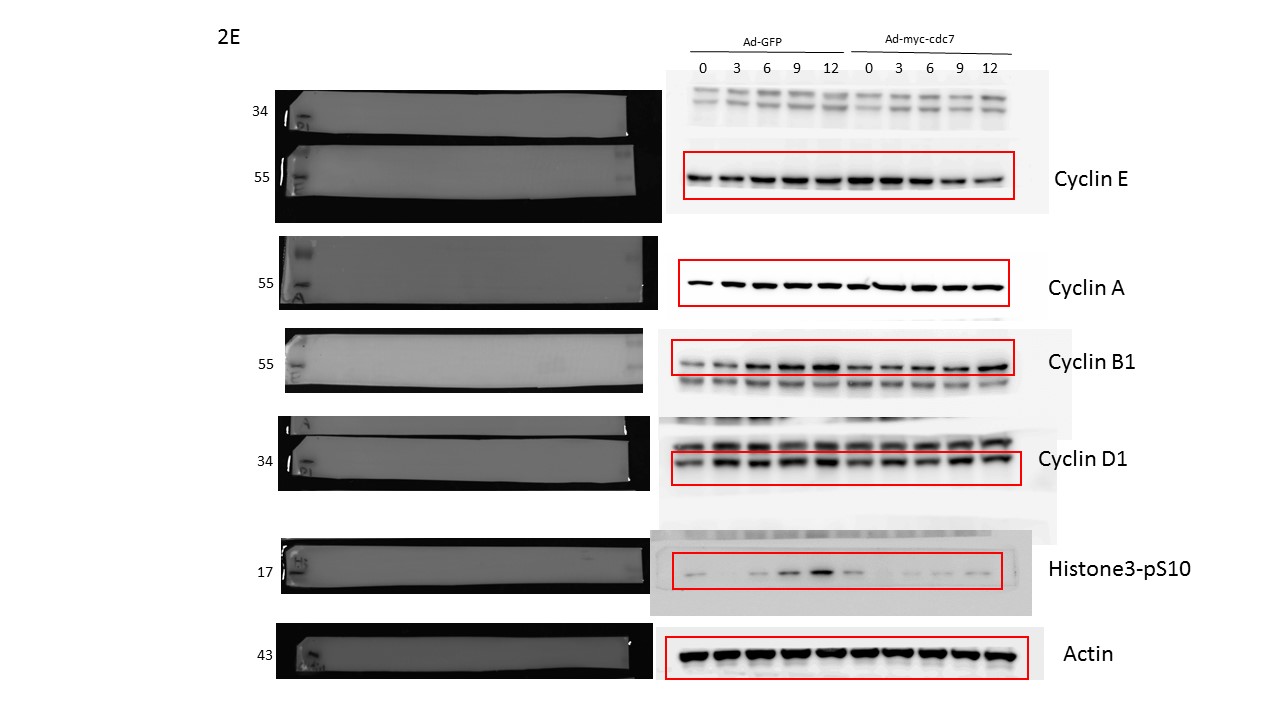

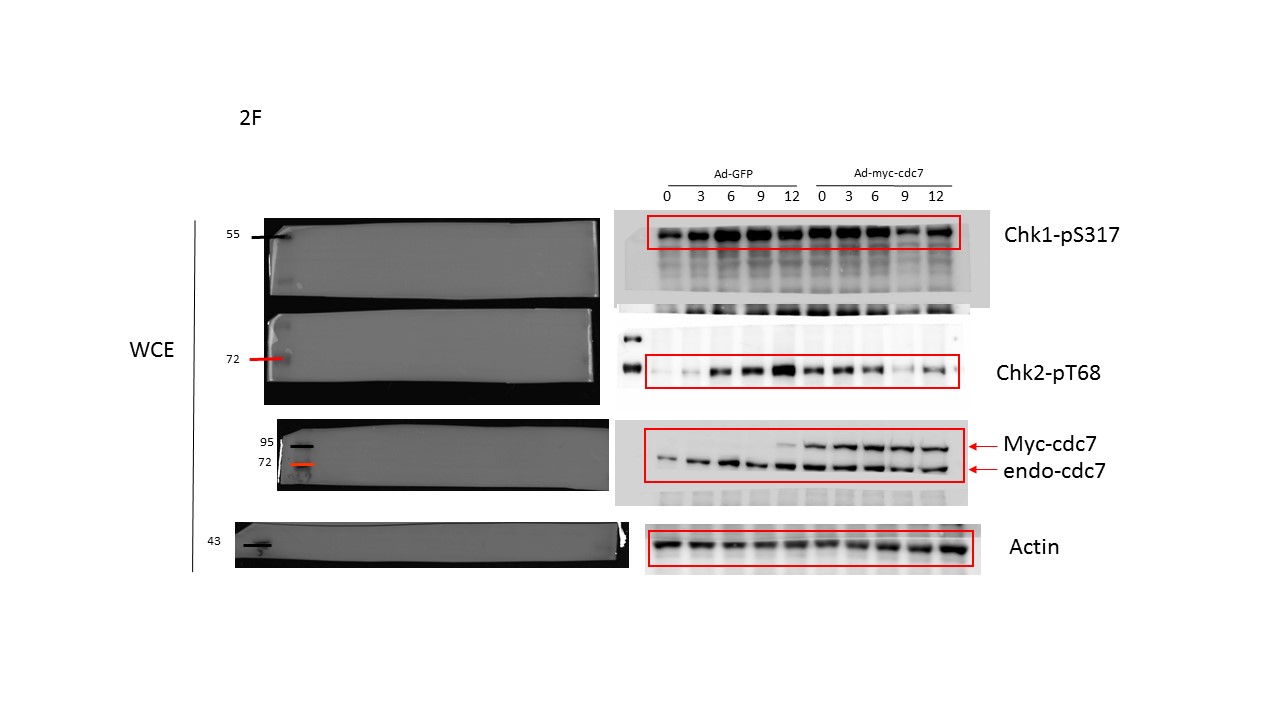

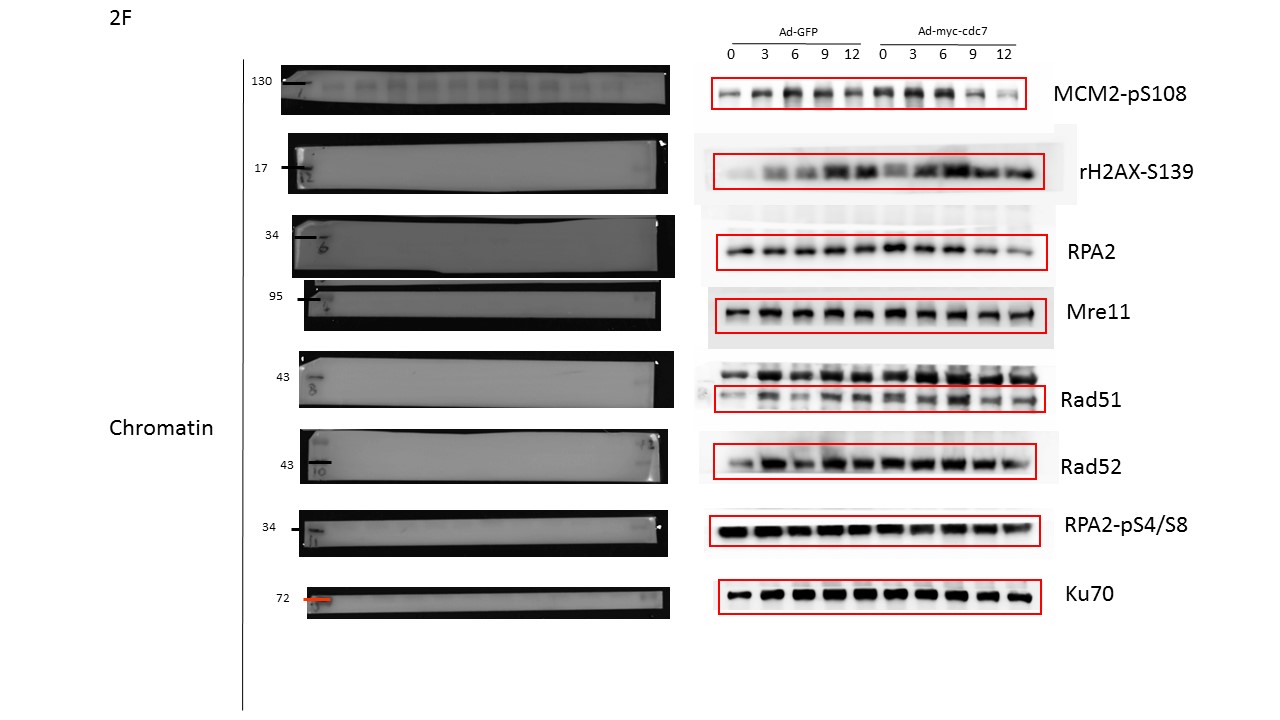

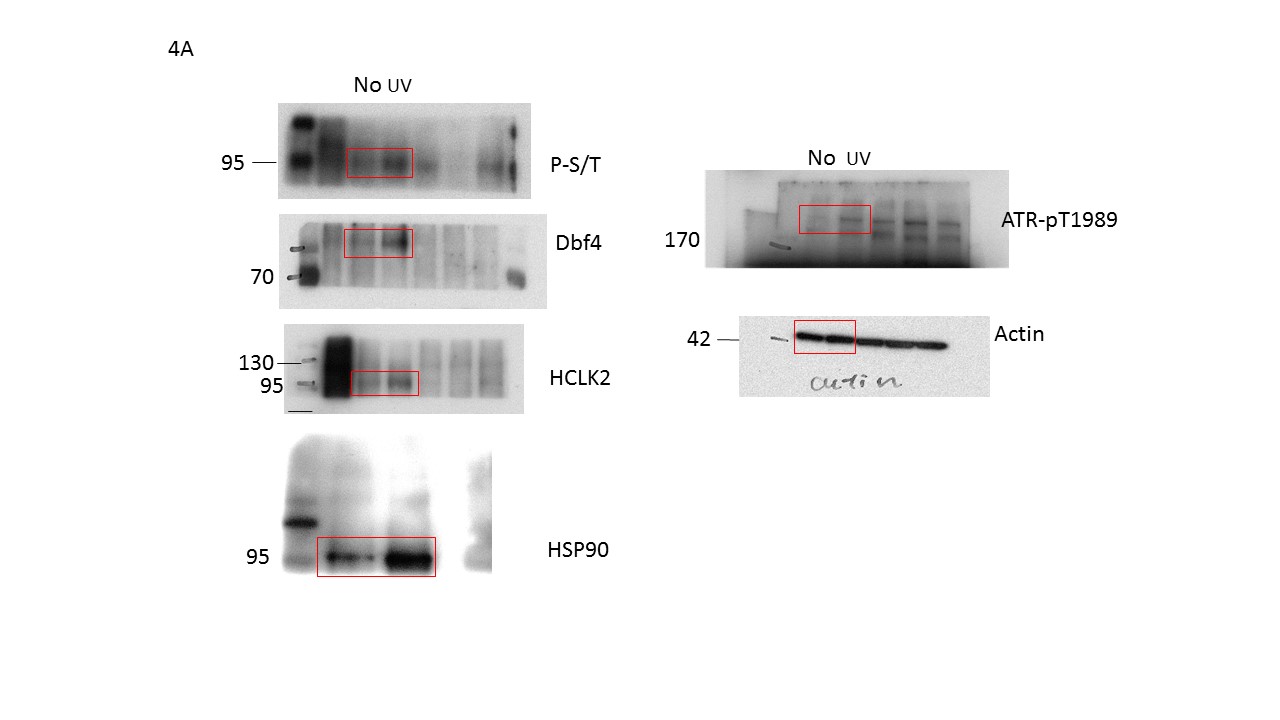

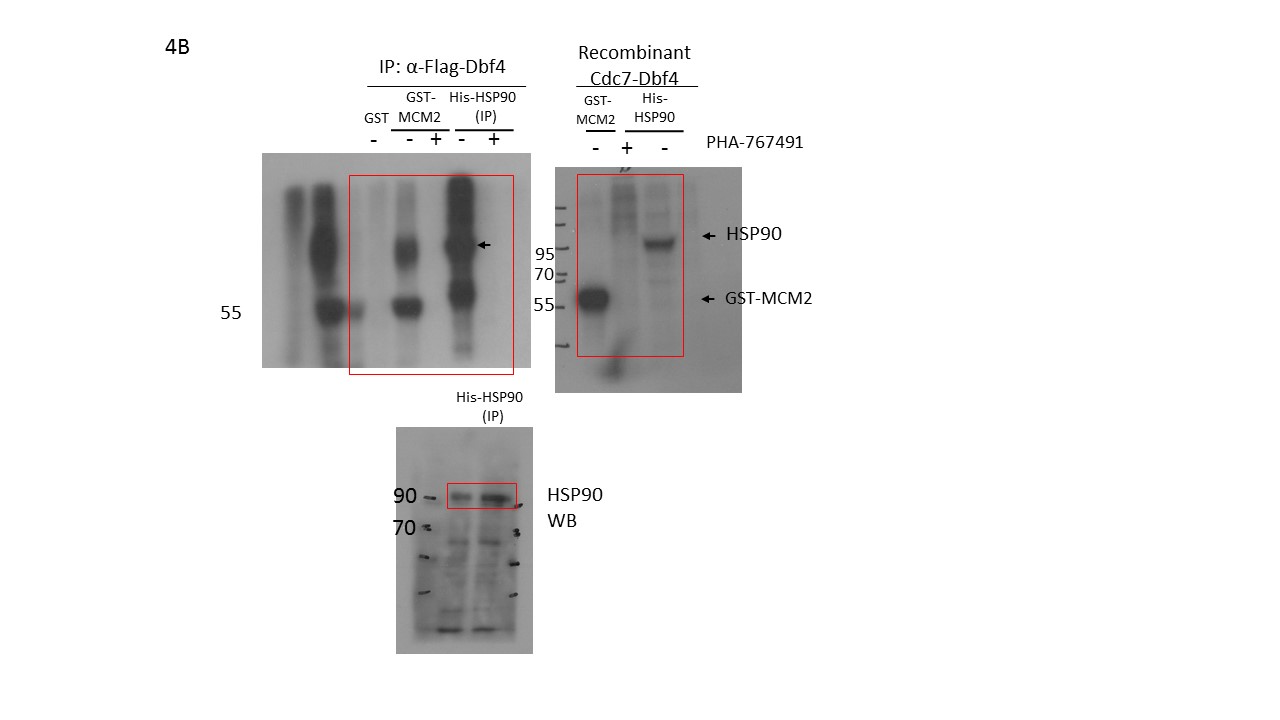

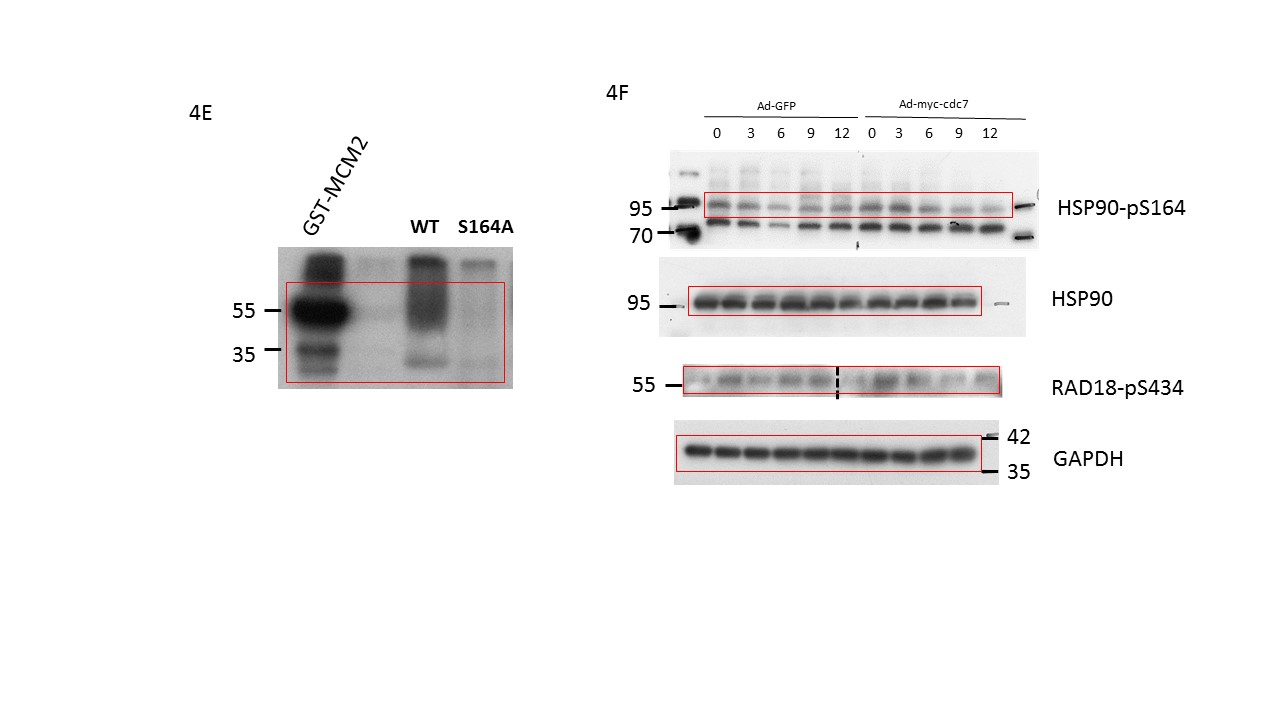
**

**
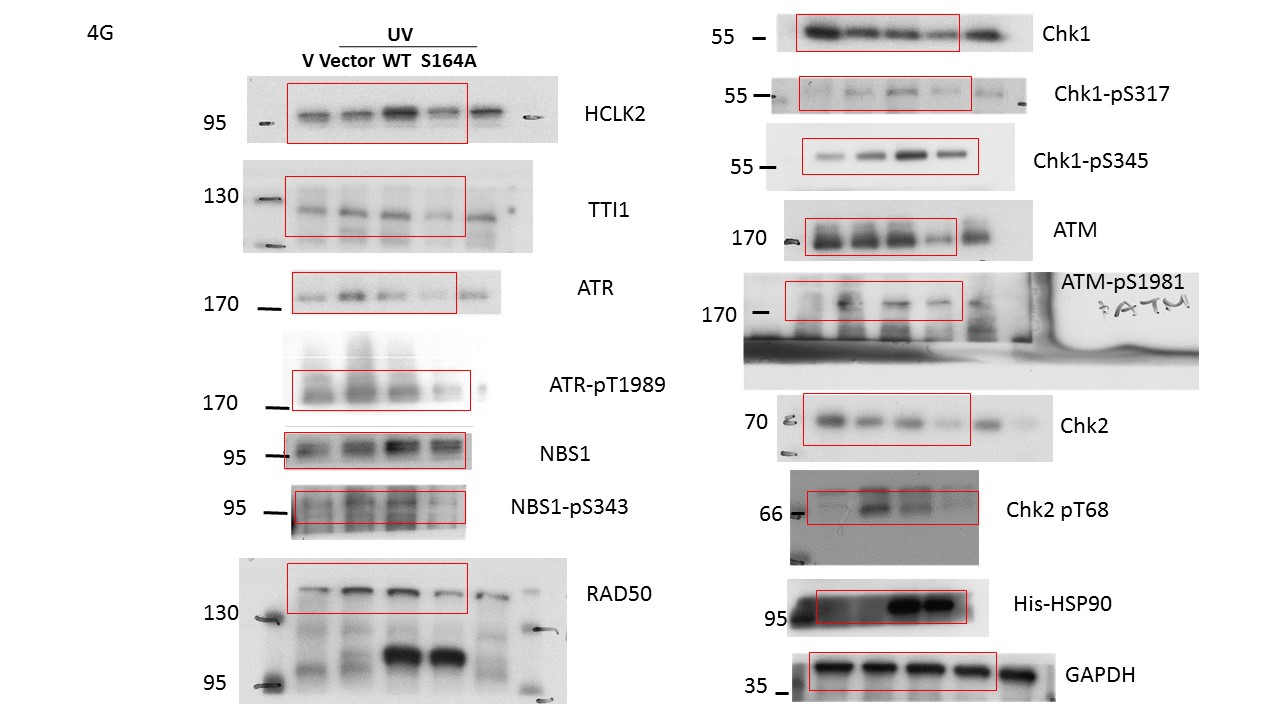
**

**
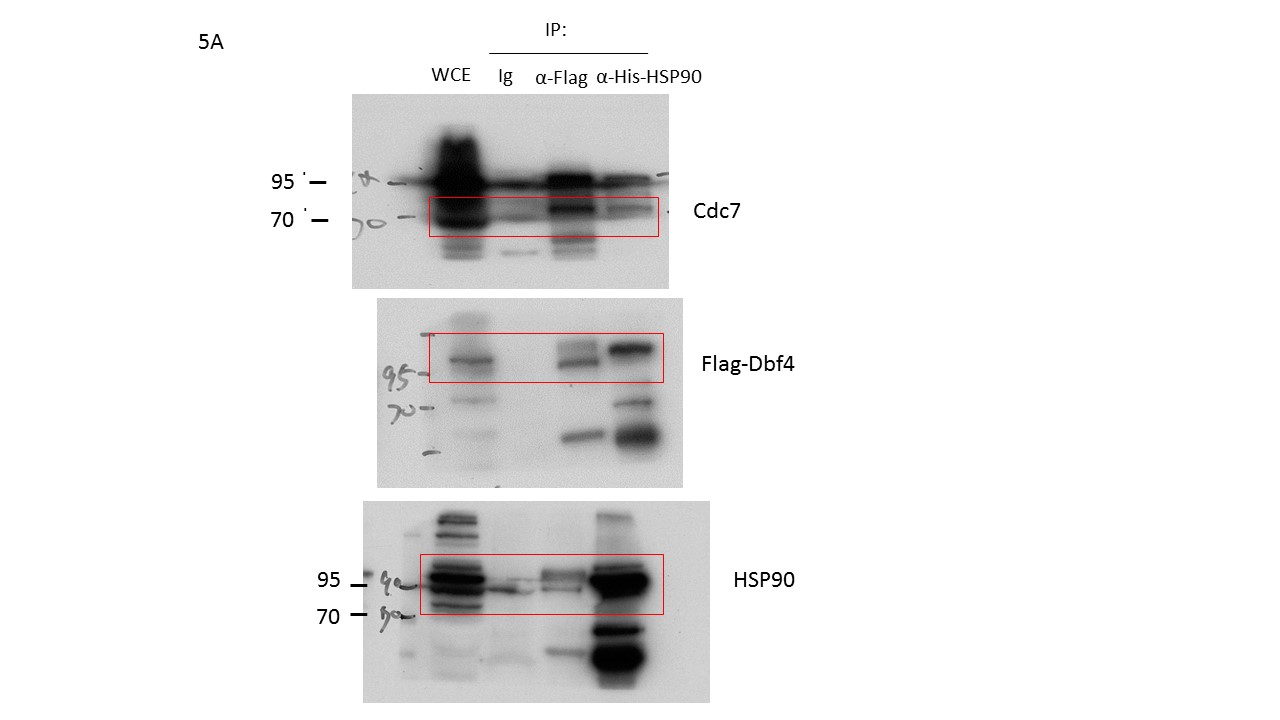

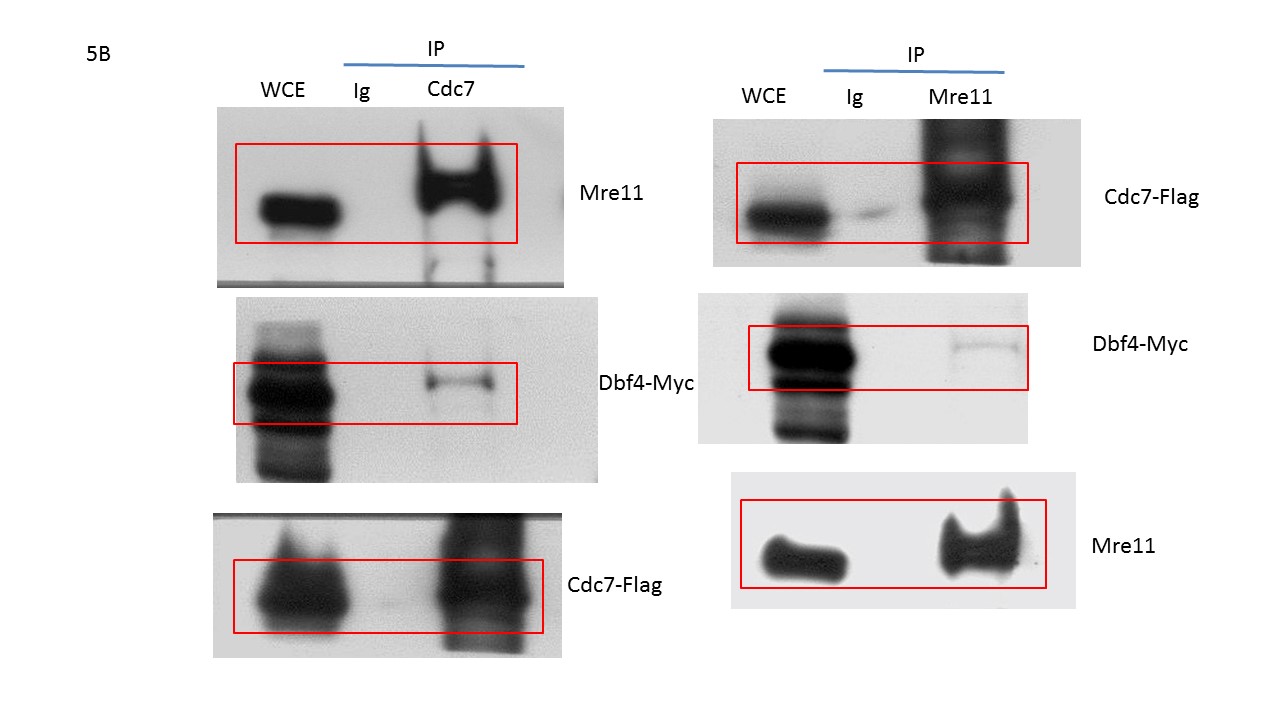

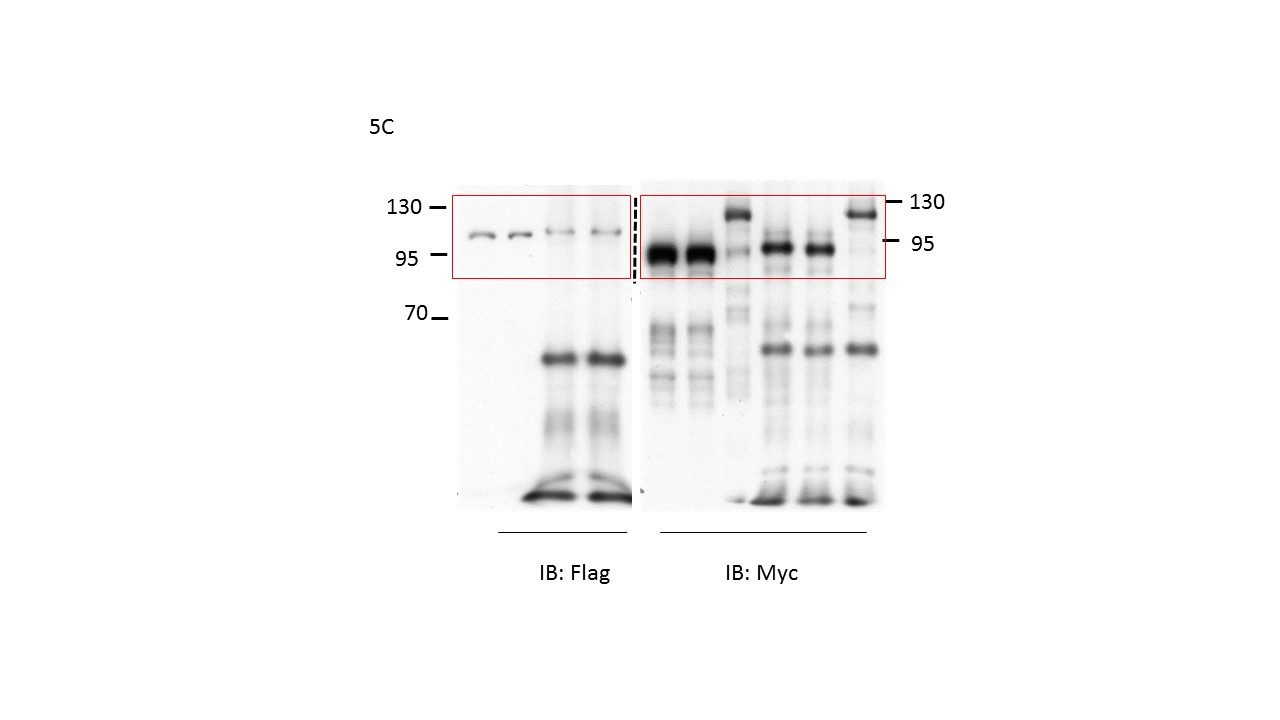

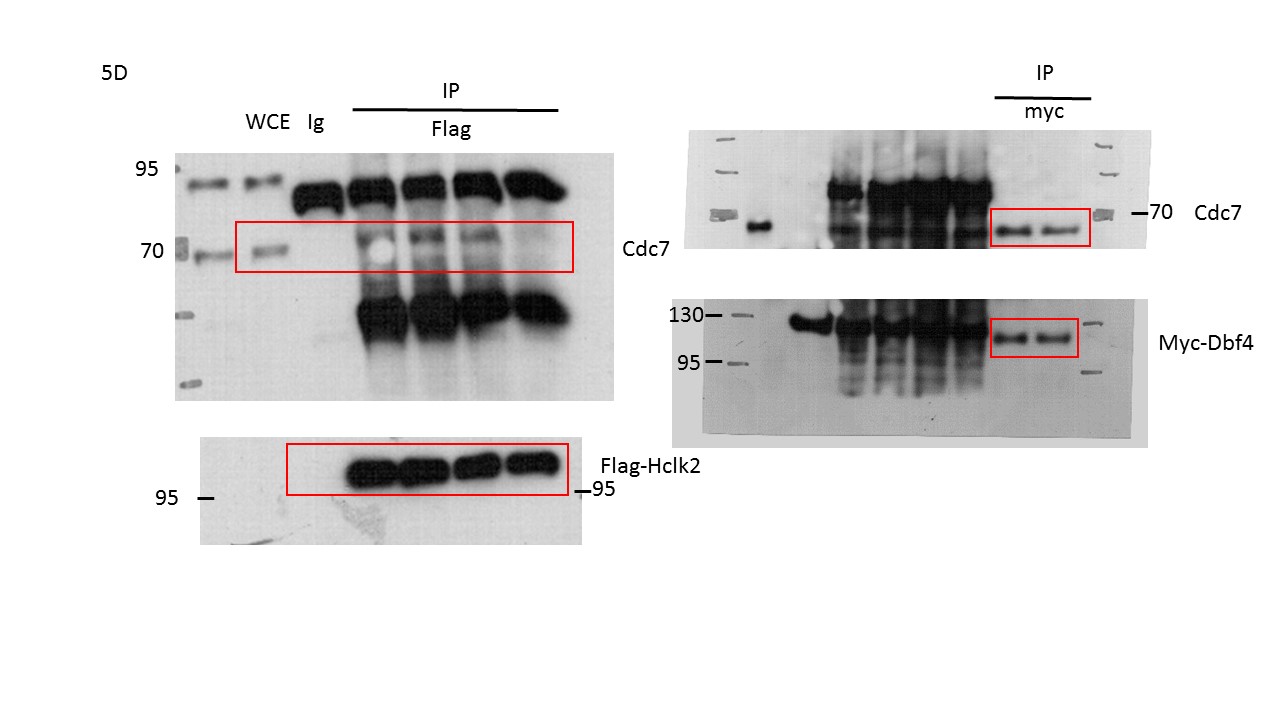

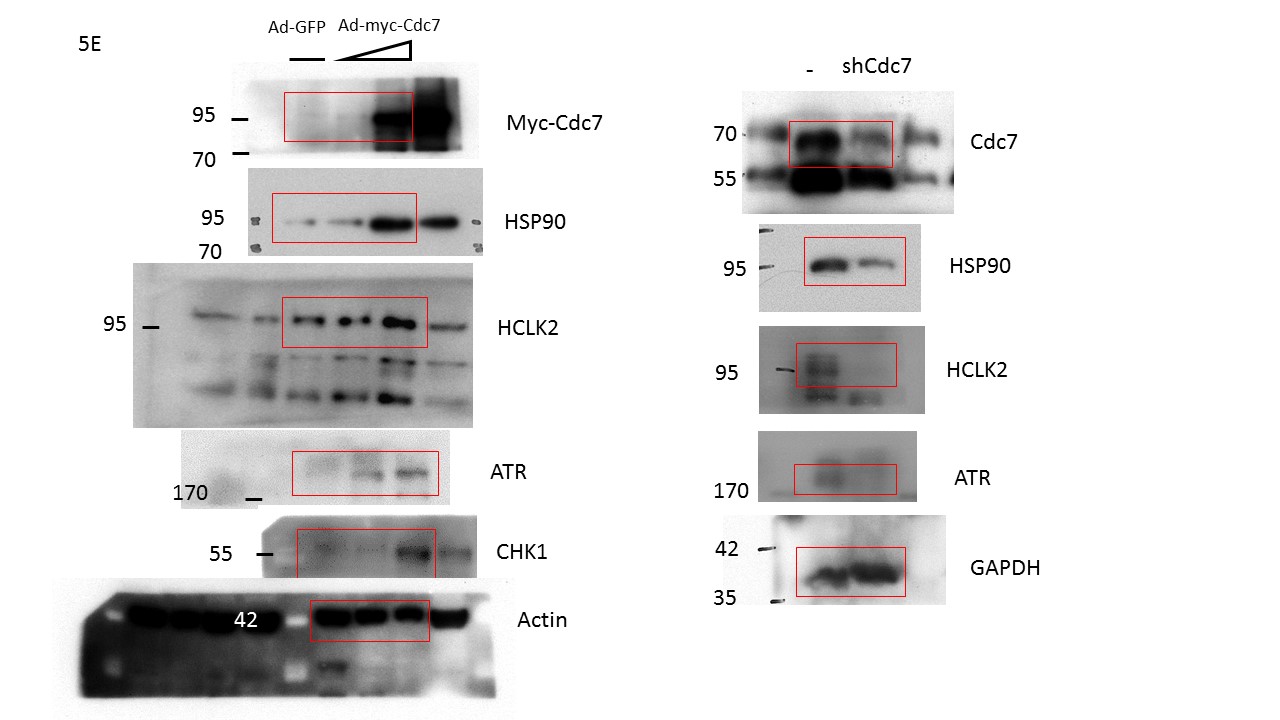
**

**
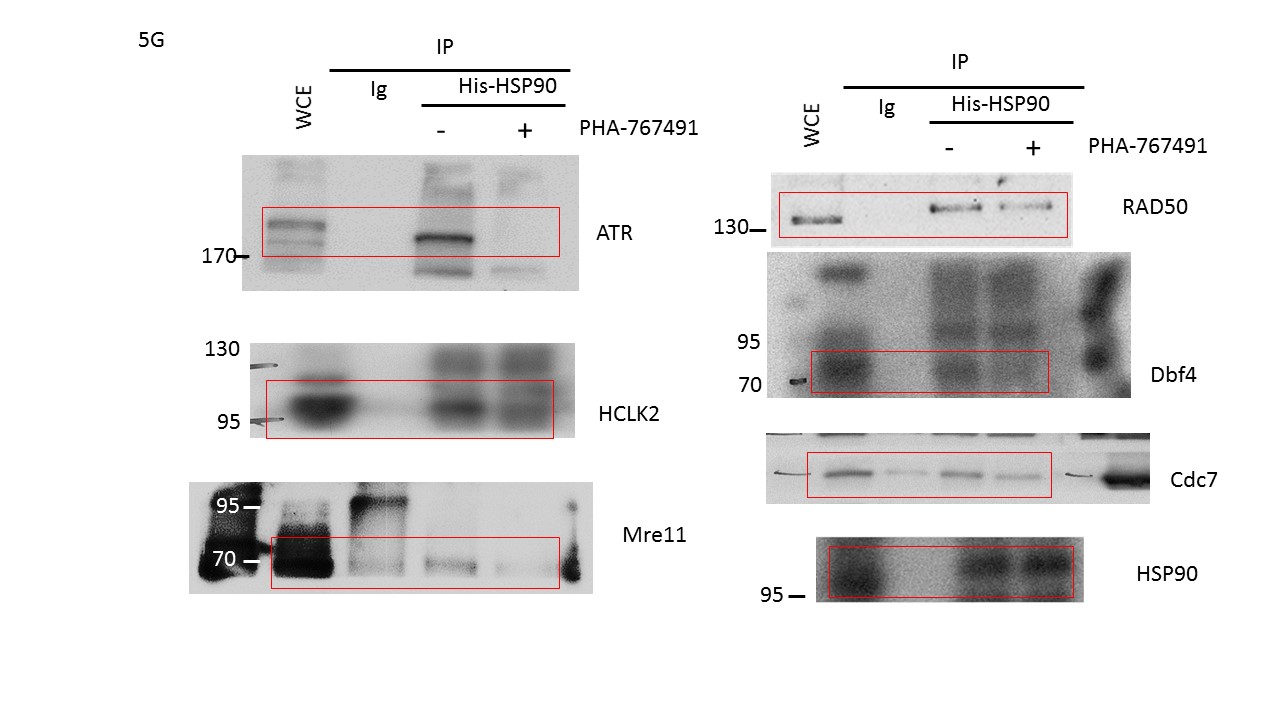
**

**
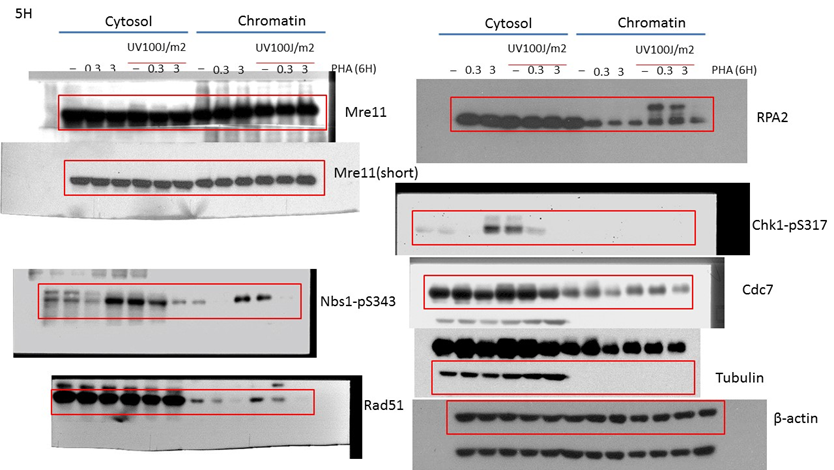
**

**
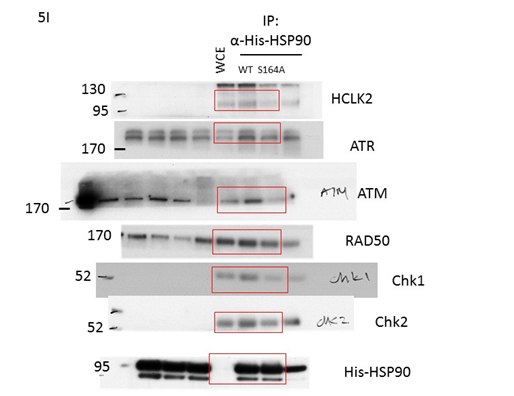
**

**
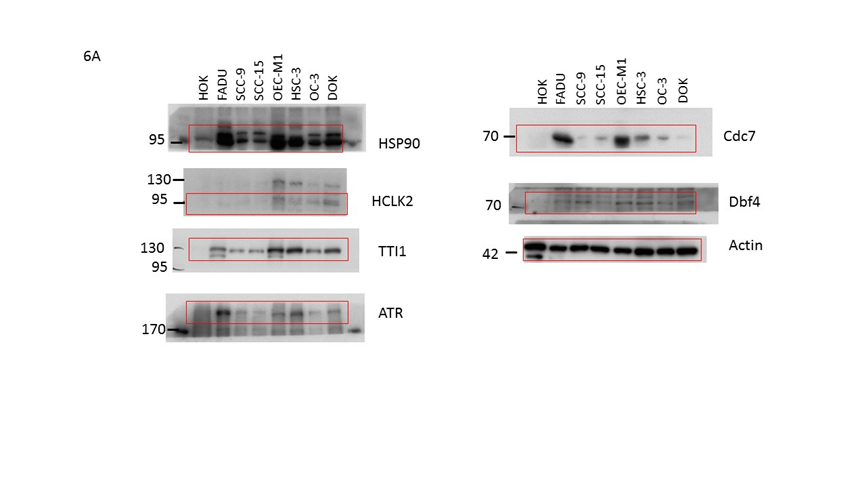
**

**Figure S8.** **The uncropped and unprocessed scans of original blots shown in figures in this study.**
